# Inhibition of miR-148a-3p Ameliorates Prion Accumulation by Restoring ATP6AP2-Mediated Lysosomal Acidification

**DOI:** 10.3390/ijms27219524

**Published:** 2026-10-24

**Authors:** Rui Xu, Cao Chen, Tong-Xin Sun, Ju-Zheng Li, Xing-Hao Zhai, Bing Xu, Ru-Han A, Xue-Qin Zhang, Fang-Fan Ning, Dong-Lin Liang, Run-Dong Cao, Wei Zhou, Li-Ping Gao, Kang Xiao, Qi Shi, Xiao-Ping Dong

**Affiliations:** 1 National Key Laboratory of Intelligent Tracking and Forecasting for Infectious Diseases, NHC Key Laboratory for Medical Virology and Viral Diseases, Beijing Key Laboratory of Viral Infectious Diseases, National Institute for Viral Disease Control and Prevention, Chinese Center for Disease Control and Prevention & Chinese Academy of Preventive Medicine, Beijing 102206, China; 2 Shanghai Institute of Infectious Disease and Biosafety, Shanghai 200003, China

**Keywords:** prion disease, miR-148a-3p, ATP6AP2, lysosomal acidification, autophagic flux, PrP^Sc^

## Abstract

Prion diseases (PrDs) are fatal neurodegenerative disorders characterized by the accumulation of misfolded prion protein (PrP^Sc^) and progressive impairment of protein quality-control systems. Lysosomal dysfunction and defective autophagic flux contribute to PrP^Sc^ accumulation, but the upstream regulatory mechanisms remain incompletely defined. In this study, we investigated the regulatory role of miR-148a-3p during prion infection. We found that miR-148a-3p expression is markedly increased in ME7- and 139A-infected mouse brains and prion-infected SMB-S15 cells. Dual-luciferase assays confirmed that miR-148a-3p directly targets the PRNP 3′UTR, negatively regulating cellular PrP expression. In prion-infected cells, inhibition of miR-148a-3p reduced proteinase K (PK)-resistant PrP (PrP^res^), delayed RT-QuIC conversion, and decreased PMCA-amplified PrP^res^. Through bioinformatic prediction and experimental validation, we identified ATP6AP2, a V-ATPase-associated regulator of lysosomal acidification, as a high-confidence target of miR-148a-3p. ATP6AP2 expression was significantly decreased in sCJD transcriptomic data, scrapie-infected mouse brains, and prion-infected cells. Importantly, inhibition of miR-148a-3p restored ATP6AP2 expression, which improved lysosomal acidification and alleviated impaired autophagic flux. Furthermore, overexpression of ATP6AP2 directly reduced PrP^Sc^ accumulation in SMB-S15 cells and diminished prion seeding capacities in RT-QuIC and PMCA assays. These findings identify miR-148a-3p as an upstream regulator of ATP6AP2-dependent lysosomal dysfunction in prion infection. Inhibition of miR-148a-3p may restore lysosomal clearance capacity and reduce PrP^Sc^ accumulation, suggesting a potential regulatory strategy for PrDs.

## 1. Introduction

Prion diseases (PrDs), or transmissible spongiform encephalopathies (TSEs), comprise a devastating group of fatal and rapidly progressive neurodegenerative disorders. Prominent examples include Creutzfeldt–Jakob disease (CJD) in humans, scrapie in sheep, bovine spongiform encephalopathy (BSE) in cattle, and chronic wasting disease (CWD) in cervids [1,2,3,4]. These conditions are defined by extensive spongiform vacuolation, neuronal loss, gliosis, and accumulation of pathologically misfolded prion protein in the central nervous system (CNS). The fundamental molecular event in PrD pathogenesis is the conformational conversion of endogenous cellular prion protein (PrP^C^), a glycosylphosphatidylinositol (GPI)-anchored glycoprotein enriched in neuronal membranes, into the infectious and aggregation-prone isoform known as scrapie prion protein (PrP^Sc^) [5,6,7,8]. Progressive cerebral accumulation of PrP^Sc^ aggregates initiates a cascade of neurotoxic events, including neuroinflammation, synaptic impairment, and neuronal injury.

To counteract the accumulation of misfolded proteins such as PrP^Sc^, neurons depend on cellular protein quality control systems, particularly the autophagy-lysosome pathway [9,10,11]. A substantial fraction of intracellular PrP^Sc^ traffics through the endolysosomal system [12], and misfolded PrP species present in the endoplasmic reticulum (ER) or cytoplasm can be targeted for lysosomal degradation via the autophagic pathway [13,14,15]. Autophagy is therefore considered an important host defense mechanism against prion propagation [16,17]. However, during the progression of PrDs, lysosomal function and autophagic flux are frequently compromised, leading to the accumulation of enlarged or incomplete autophagic vacuoles and inefficient PrP^Sc^ clearance [9,18,19]. Defining the upstream regulatory mechanisms that impair lysosomal acidification and autophagic flux in prion infection is essential for identifying new therapeutic targets.

ATPase H^+^ transporting accessory protein 2 (ATP6AP2), originally characterized as the (pro)renin receptor, is also an accessory component required for vacuolar-type H^+^-ATPase (V-ATPase) function [20]. Increasing evidence links aberrant ATP6AP2 expression or function in the CNS to neuropathological and neurodegenerative phenotypes [21,22]. Mutations or conditional depletion of ATP6AP2 have been associated with cognitive impairment, epilepsy, parkinsonism-related phenotypes, synaptic abnormalities, and neuronal degeneration [23,24]. Consistent with these clinical observations, conditional knockout of ATP6AP2 causes profound neurological impairments, manifesting as photoreceptor vacuolation and memory deficits in fruit flies, and abnormal fear memory, synaptic disorganization, and cortical–hippocampal neurodegeneration in mice [25]. Mechanistically, ATP6AP2 contributes to lysosomal acidification by supporting V-ATPase assembly and activity, which are required for lysosomal hydrolase maturation and autophagosome–lysosome fusion [26]. Loss or reduction in ATP6AP2 can therefore impair lysosomal acidity and autophagic degradation, favoring the accumulation of undegraded substrates [27]. However, whether ATP6AP2 participates in lysosomal dysfunction and pathogenic PrP accumulation in PrDs has not been well addressed.

MicroRNAs (miRNAs) are endogenous, non-coding, single-stranded RNAs of approximately 18–22 nucleotides that regulate gene expression post-transcriptionally through the RNA-induced silencing complex (RISC) [28]. The miR-148a family has been reported to be dysregulated in neurodegenerative and neuroinflammatory contexts, including Alzheimer’s disease (AD) and Parkinson’s disease (PD), often correlating with disease severity [29,30,31]. Mechanistically, miR-148a-3p has been implicated in neuronal injury, neuroinflammation, DNA methylation, and synaptic regulation by targeting key factors such as PTEN, TLR4, and DNMT1 [32,33,34,35]. Nevertheless, the expression profile and functional role of miR-148a-3p in PrDs remain uncharacterized. Crucially, whether miR-148a-3p regulates PrP expression or lysosome-dependent PrP^Sc^ clearance during prion infection has not been determined.

In this study, we report a significant increase in miR-148a-3p in both prion-infected mouse brains and prion-infected cells. Bioinformatic prediction and dual-luciferase validation showed that miR-148a-3p directly targets the PRNP 3′UTR and negatively regulates cellular PrP expression in several cell lines. In prion-infected cells, miR-148a-3p inhibition partially reduced PrP^Sc^ accumulation, delayed RT-QuIC conversion, and decreased PMCA-amplified proteinase K (PK)-resistant PrP (PrP^res^). Further screening identified ATP6AP2, a V-ATPase-associated regulator of lysosomal acidification, as a high-confidence candidate target of miR-148a-3p. ATP6AP2 was decreased in prion-infected brains and cells, whereas inhibition of miR-148a-3p restored ATP6AP2 expression, improved lysosomal acidification, and alleviated impaired autophagic flux. Moreover, ATP6AP2 overexpression partially reduced PrP^Sc^ accumulation and PMCA capacity. These findings suggest that miR-148a-3p-mediated suppression of ATP6AP2 contributes to lysosomal dysfunction during prion infection and may represent a regulatory pathway for enhancing PrP^Sc^ clearance.

## 2. Results

### 2.1. Remarkable Increase in miR-148a-3p in the Brains of Prion-Infected Mouse Models and Prion-Infected Cells

**Figure 1:**
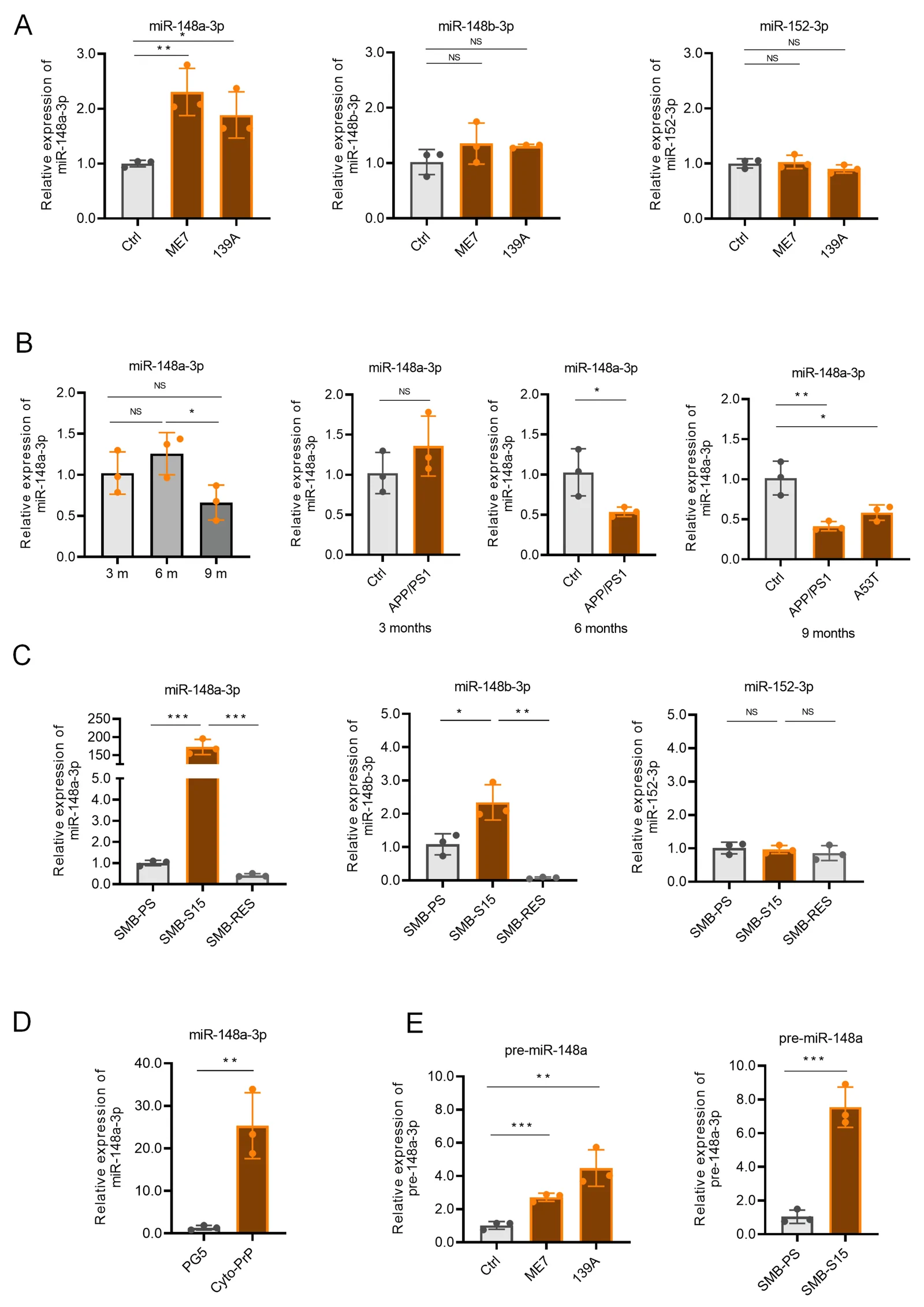
Remarkable increase in miR-148a-3p in the brains of prion-infected mouse models and prion-infected cells. (**A**) qRT-PCR assays for miR-148a-3p (**left**), miR-148b-3p (**middle**), and miR-152-3p (**right**) in brain tissues from ME7- or 139A-infected mice at the terminal stage and age-matched normal controls (Ctrl). (**B**) Relative expression of miR-148a-3p in normal mouse brains at different ages (3m, 6m, and 9m) and in transgenic mouse models of Alzheimer’s disease (APP/PS1) and Parkinson’s disease (A53T) compared with age-matched controls. (**C**) qRT-PCR assays for miR-148a-3p (**left**), miR-148b-3p (**middle**), and miR-152-3p (**right**) in prion-infected SMB-S15 cells, their normal partner SMB-PS cells, and prion-cured SMB-RES cells. (**D**) Relative expression of miR-148a-3p in HEK-293T cells transiently transfected with plasmids expressing wild-type PrP (PG5) or cytosolic PrP mutant (Cyto-PrP). (**E**) qRT-PCR assays for precursor miR-148a (pre-miR-148a) in brain tissues from ME7- or 139A-infected mice at the terminal stage and age-matched controls (**left**), and in SMB-S15 and SMB-PS cells (**right**). Relative expression levels normalized to U6 snRNA. Data are presented as the mean ± SD from at least three independent experiments. For panels A and C, *n* = 3 animals/independent experiments per group, and statistical differences were evaluated using one-way ANOVA followed by Tukey’s multiple-comparison test. For panel D and the right panel of E, n = 3 independent experiments, and a two-tailed Student’s *t*-test was used. For the left panel of E, one-way ANOVA followed by Tukey’s multiple-comparison test was used. For panel B, the three age groups were analyzed using one-way ANOVA followed by Tukey’s multiple-comparison test, whereas disease-model groups were compared with age-matched controls using a two-tailed Student’s *t*-test. * *p* < 0.05, ** *p* < 0.01, *** *p* < 0.001, NS, not significant.

To assess the potential changes in miR-148a-3p, miR-148b-3p and miR-152-3p during prion infection, total RNA was prepared from the brains of two scrapie-infected mouse models, ME7- and 139A-infected mice at terminal stage, and subjected to qRT-PCR analysis. Markedly higher levels of miR-148a-3p were detected in the brains of ME7- and 139A-infected mice, whereas the levels of miR-148b-3p and miR-152-3p were comparable to those in age-matched controls (Figure 1A). We also evaluated brain miR-148a-3p levels in normal mice of different ages and in transgenic mouse models of AD and PD. miR-148a-3p levels were comparable between 3- and 6-month-old normal mice, with a modest but statistically significant reduction at 9 months (Figure 1B, left). In contrast, brain miR-148a-3p was significantly decreased in 6- and 9-month-old AD transgenic mice (APP/PS1), and in the 9-month-old PD transgenic mice (A53T) compared with age-matched controls (Figure 1B, right three panels).

qRT-PCR analysis of miR-148a-3p, miR-148b-3p and miR-152-3p in prion-infected cultured cells also revealed a similar pattern to that observed in prion-infected animals. miR-148a-3p was significantly increased in the prion-infected cell line SMB-S15 compared with its uninfected partner cell line SMB-PS, whereas miR-148b-3p showed a slight but statistically significant increase and miR-152-3p remained nearly unchanged (Figure 1C). In the prion-cured SMB-RES cells, in which prion propagation was removed by resveratrol treatment, the increased levels of miR-148a-3p and miR-148b-3p were markedly reduced (Figure 1C). To test whether cytoplasmic PrP accumulation influences miR-148a-3p expression, HEK-293T cells were transiently transfected with plasmids expressing wild-type PrP (PG5-PrP) or cytosolic PrP (Cyto-PrP). miR-148a-3p levels were significantly higher in cells expressing Cyto-PrP than in cells expressing PG5-PrP (Figure 1D). In addition, precursor of miR-148a (pre-miR-148a) levels were increased in the brains of ME7- and 139A-infected mice at terminal stage, and in SMB-S15 cells (Figure 1E).

### 2.2. miR-148a-3p Directly Targets the 3′UTR of the PRNP

**Figure 2:**
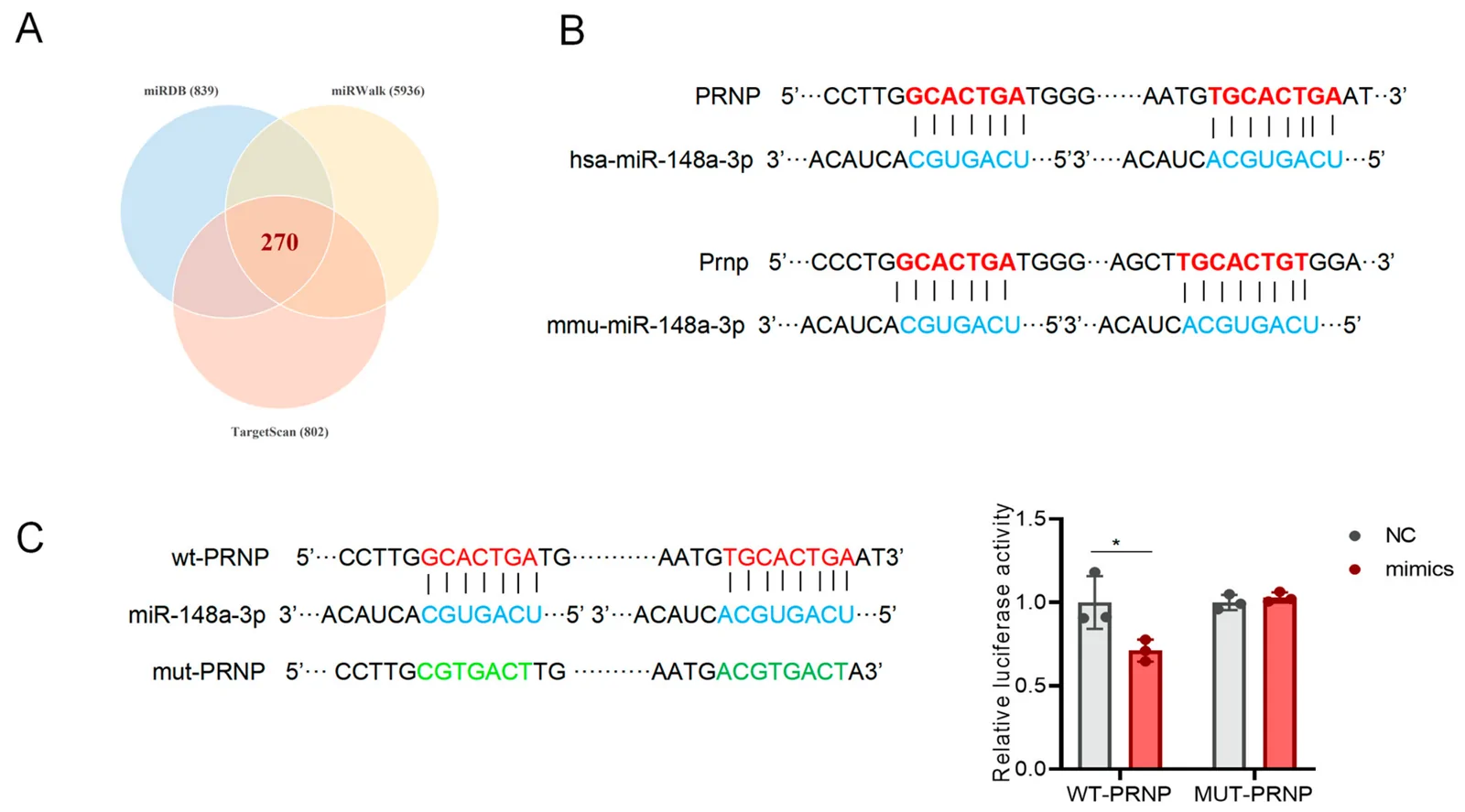
miR-148a-3p directly targets the 3′UTR of *PRNP*. (**A**) Bioinformatic prediction of miR-148a-3p targets. The Venn diagram illustrates the intersection of predicted target genes from three independent databases (miRDB, TargetScan, and miRWalk), yielding 270 common candidate genes including *PRNP*. (**B**) Sequence alignment of miR-148a-3p with the predicted binding sites in the 3′UTR of human *PRNP* and mouse *Prnp*. The red sequences indicate the predicted target regions within the PRNP/Prnp 3′UTR, whereas the blue sequences indicate the complementary seed region of miR-148a-3p. The conserved seed-matching nucleotides support the potential interaction between miR-148a-3p and *PRNP*/*Prnp*. (**C**) Schematic design and validation layout of the dual-luciferase reporter assay for the predicted miR-148a-3p binding sites within the PRNP 3′UTR. The left panel shows the sequence design of the wild-type PRNP 3′UTR reporter (wt-PRNP) and the corresponding mutant reporter (mut-PRNP). The predicted miR-148a-3p seed-matching nucleotides in the PRNP 3′UTR are highlighted in red, the complementary nucleotides in miR-148a-3p are highlighted in blue, and the substituted nucleotides introduced into the mutant reporter are highlighted in green. The right panel shows the relative luciferase activity of HEK-293T cells co-transfected with miR-148a-3p mimics or negative control (NC) together with wt-PRNP or mut-PRNP reporter plasmids. Relative luciferase activity was calculated by normalizing firefly luciferase activity to Renilla luciferase activity. Data are presented as the mean ± SD from three independent experiments. For panel C, *n* = 3 independent experiments, and statistical differences were evaluated using one-way ANOVA followed by Tukey’s multiple-comparison test. * *p <* 0.05.

Using three different online databases, miRDB, TargetScan, and miRWalk, we identified 270 consensus potential target genes of miR-148a-3p, including the PRNP gene (Figure 2A). Two putative binding sites were predicted in the human PRNP 3′UTR, corresponding to nt. 138-144 and nt. 1432-1439, and two conserved sites were predicted in the mouse Prnp 3′UTR, corresponding to nt. 133-139 and nt. 930-937, respectively (Figure 2B and Supplementary Figure S1A–C). To validate whether PRNP is a direct target of miR-148a-3p, a dual-luciferase reporter assay was conducted. Reporter plasmids containing either the wild-type PRNP 3′UTR encompassing the predicted binding sites or a mutant 3′UTR with disrupted seed-matching sequences were constructed (Figure 2C, left). Co-transfection of the WT-PRNP reporter with miR-148a-3p mimics in HEK-293T cells reduced relative luciferase activity compared with the negative control (NC). This inhibitory effect was abolished when the mut-PRNP reporter was utilized, restoring the luciferase activity to the basal level (Figure 2C, right).

### 2.3. miR-148a-3p Downregulates PrP Expression in Several Culture Cell Lines

**Figure 3:**
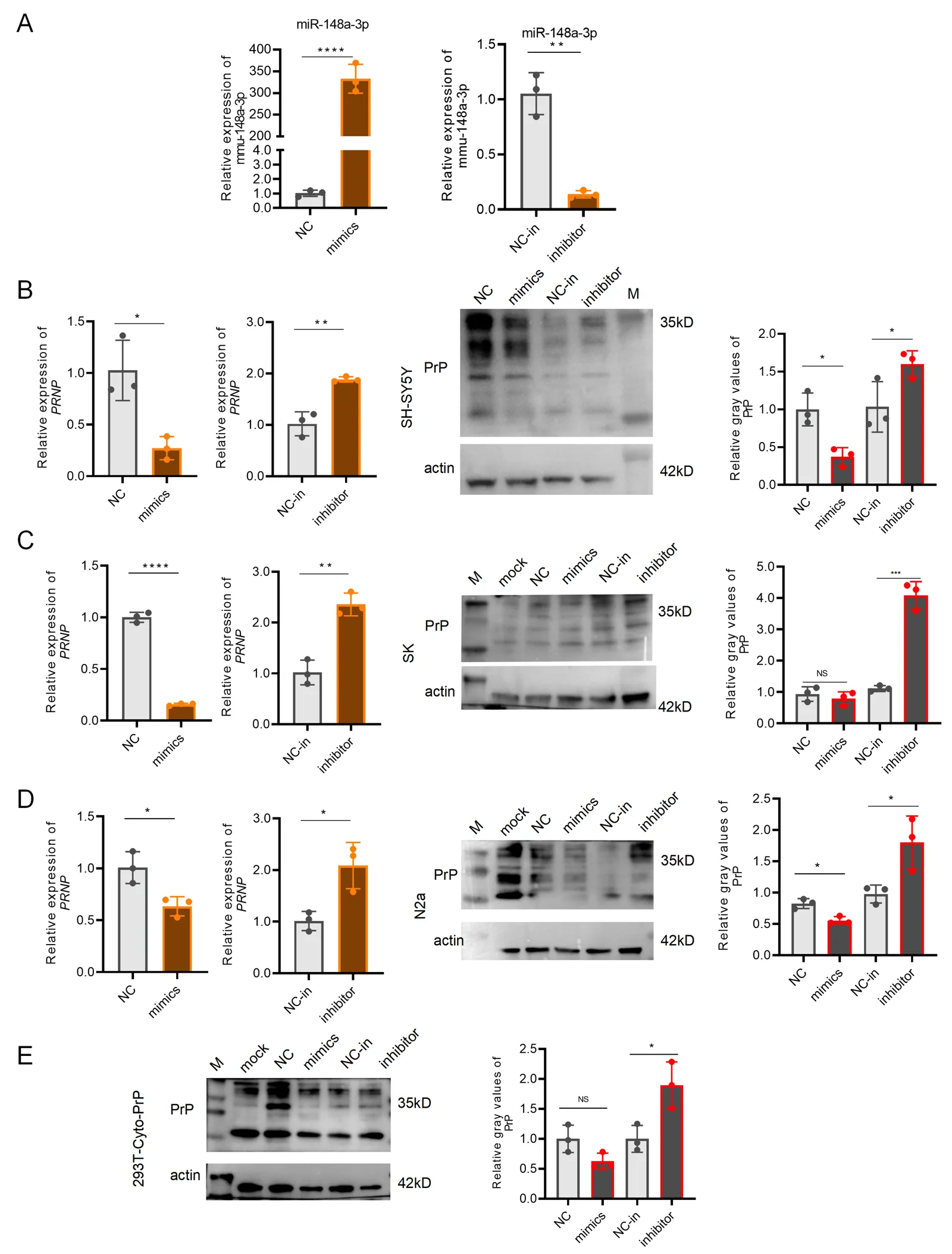

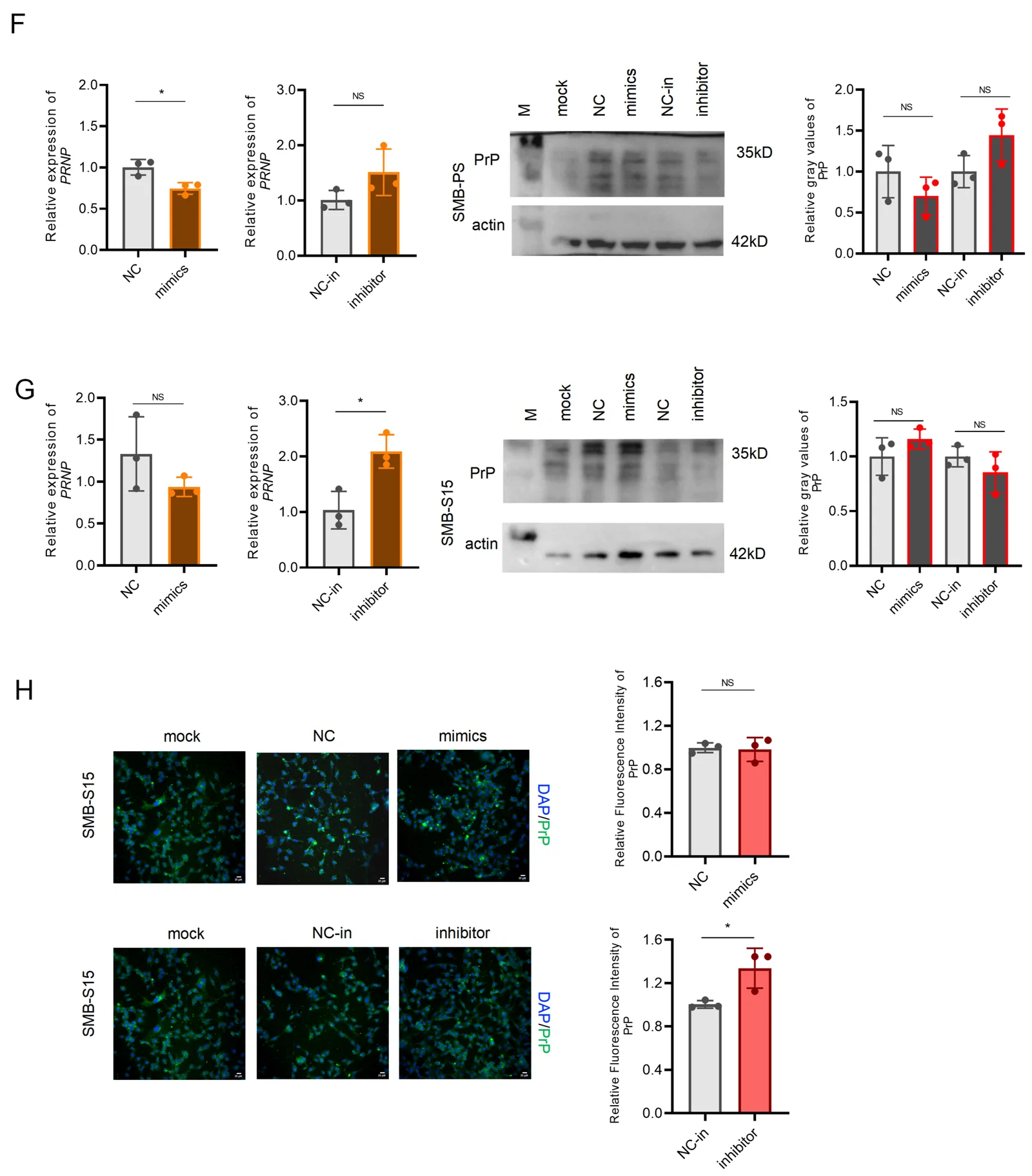
miR-148a-3p negatively regulates cellular PrP expression in multiple cell lines. (**A**) Verification of transfection efficiency in SMB-S15 cells. Relative miR-148a-3p levels in cells transiently transfected with miR-148a-3p mimics (**left**) or inhibitor (**right**), together with their respective negative controls (NC, and the negative control for inhibitor, denoted as NC-in), were measured by qRT-PCR. (**B**–**D**) Transcriptional and protein expression levels of endogenous PrP after miR-148a-3p manipulation in SH-SY5Y cells (**B**), SK-N-SH cells (**C**), and N2a cells (**D**). The left panels show relative PRNP/Prnp mRNA levels, and the right panels show representative Western blots of PrP and corresponding densitometric analyses. (**E**) Western blots and quantitative analysis of PrP expression in HEK-293T cells transiently expressing cytosolic PrP mutant (Cyto-PrP) after co-transfection with miR-148a-3p mimics, inhibitor, or their controls. (**F**,**G**) PrP mRNA and protein levels in SMB-PS cells (**F**) and SMB-S15 cells (**G**) after transfection with miR-148a-3p mimics or inhibitor. (**H**) Representative PrP-specific immunofluorescence images of SMB-S15 cells treated with miR-148a-3p mimics or inhibitor. PrP is shown in green, and nuclei were counterstained with DAPI (blue). Quantitative analyses of relative PrP fluorescence intensity are shown on the right. Relative mRNA expression levels were normalized to GAPDH or U6. Data are presented as the mean ± SD from at least three independent experiments. For panel A, *n* = 3 independent experiments, and statistical differences were evaluated using a two-tailed Student’s t-test. For panels B–H, *n* = 3 independent experiments per group, and statistical differences were evaluated using one-way ANOVA followed by Tukey’s multiple-comparison test. * *p* < 0.05, ** *p* < 0.01, **** *p* < 0.0001; NS, not significant.

To explore the effects of miR-148a-3p on the expression of cellular PrP, four small RNAs were synthesized, including miR-148a-3p mimics and the corresponding negative control (NC), miR-148a-3p inhibitor and its negative control (NC-in). qRT-PCR verified that miR-148a-3p levels were markedly upregulated in cells transfected with mimics and significantly downregulated in cells transfected with inhibitor compared with their corresponding controls (Figure 3A). CCK-8 assays showed that cell viability was not significantly affected at 24, 48, or 72 h after transfection with miR-148a-3p mimics, inhibitor, or their respective negative controls (Supplementary Figure S2A), indicating no overt cytotoxicity under the transfection conditions used. The transcript and protein levels of endogenous PrP after miR-148a-3p manipulation were then measured by qRT-PCR and Western blotting in human neuroblastoma SH-SY5Y cells (Figure 3B), SK-N-SH cells (Figure 3C), and mouse neuroblastoma N2a cells (Figure 3D). miR-148a-3p mimics generally decreased PrP mRNA and protein expression, whereas miR-148a-3p inhibitor increased PrP expression. The reduction in PrP protein levels reached statistical significance in SH-SY5Y and N2a cells but not in SK-N-SH cells. Similar trends were observed in 293T cells expressing Cyto-PrP, although the decrease following miR-148a-3p mimic treatment did not reach statistical significance (Figure 3E).

miR-148a-3p mimics, inhibitor, and their corresponding controls were also introduced into SMB cells. In SMB-PS cells, miR-148a-3p mimics significantly decreased PrP mRNA levels, with a similar but non-significant trend at the protein level, whereas miR-148a-3p inhibitor induced non-significant increasing trends in PrP mRNA and protein levels (Figure 3F). In the prion-infected cell line SMB-S15, miR-148a-3p mimics and inhibitor induced similar directional changes in PrP transcripts, but had little effect on total PrP protein levels (Figure 3G). PrP-specific IFAs in SMB-S15 cells transfected with miR-148a-3p mimics also showed similar overall PrP signal intensities compared with the corresponding controls (Figure 3H). PrP-specific IFAs in SMB-S15 cells transfected with miR-148a-3p inhibitor showed significantly increased PrP signal intensity compared with NC-in (Figure 3H). Immunofluorescence may be more sensitive to changes in PrP subcellular distribution or accessible epitope availability, whereas Western blotting primarily reflects total protein abundance in whole-cell lysates; the different sensitivities and detection principles of these two methods may account for the observed difference.

These data indicate that miR-148a-3p negatively regulates PrP expression in several cell lines without prion infection, whereas PrP protein expression in prion-infected SMB-S15 cells is less responsive to miR-148a-3p manipulation.

### 2.4. Inhibition of miR-148a-3p Partially Reduces PrP^Sc^ Accumulation and Prion Seed/Replication Activity in Prion-Infected Cells

**Figure 4:**
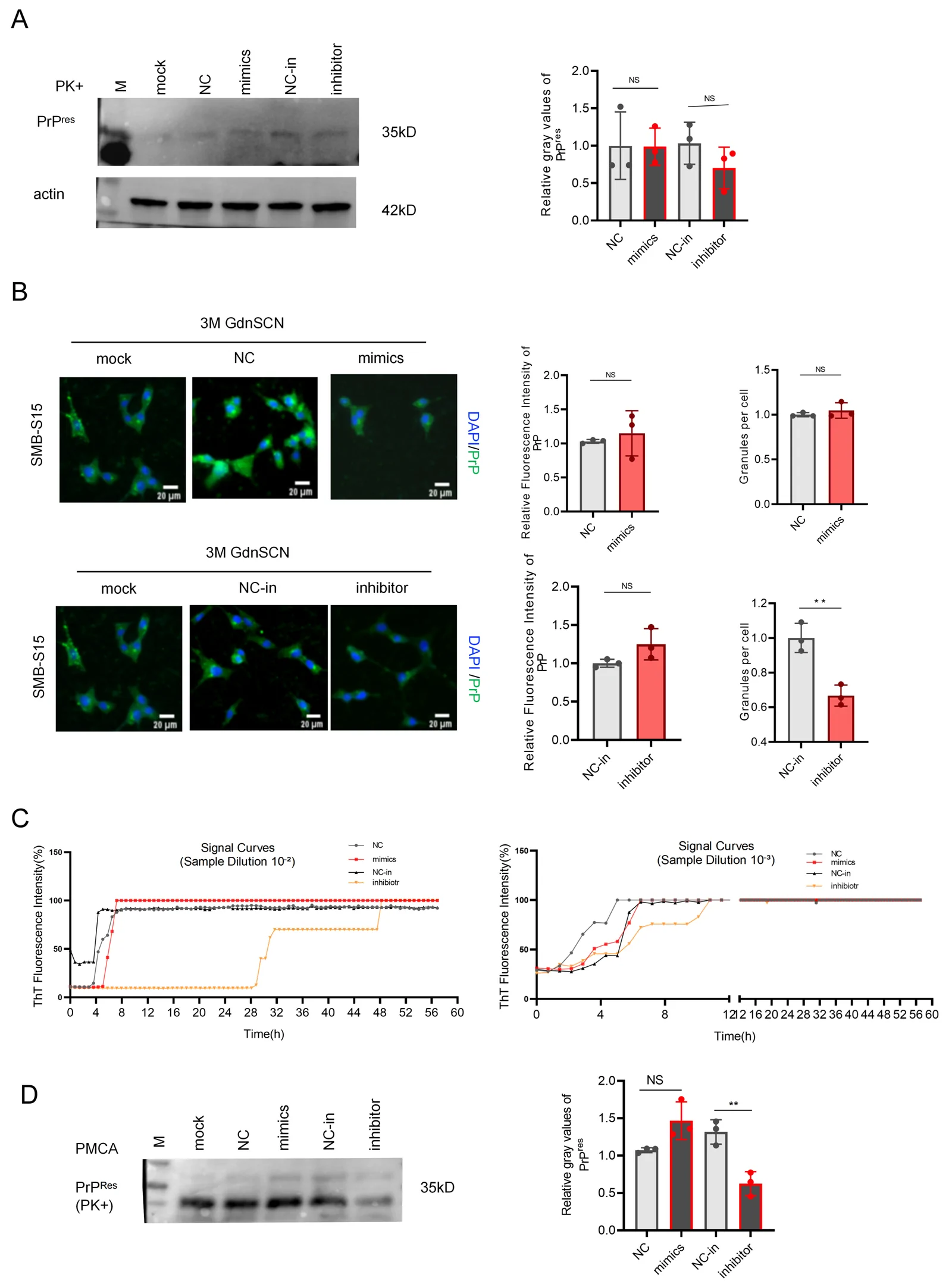
Inhibition of miR-148a-3p partially reduces PrP^Sc^ accumulation and prion seed/replication activity in prion-infected cells. (**A**) PK-digested Western blot analysis of PrP^Sc^ levels in SMB-S15 cells. Cell lysates harvested after transfection with miR-148a-3p mimics, inhibitor, or their respective negative controls (NC, and the negative control for inhibitor, denoted as NC-in) were subjected to PK digestion (20 µg/mL). Quantitative analysis of relative gray values of PrP^Sc^ is shown on the right. (**B**) Representative PrP-specific immunofluorescence assays in SMB-S15 cells. Prior to antibody incubation, cells were pre-treated with 3 M GdnSCN to denature normal PrP^C^ and expose PrP^Sc^-associated epitopes. Cells were transfected with mimics (**upper panel**) or inhibitor (**lower panel**). PrP is shown in green, and nuclei were counterstained with DAPI (blue). Scale bar, 20 μm. Quantitative analyses of relative PrP fluorescence intensity and the number of PrP-positive granules per cell are depicted on the right. (**C**) RT-QuIC assays evaluating the seeding activity of cell lysates. Lysates from SMB-S15 cells treated with mimics, inhibitor, or controls were subjected to 10-fold serial dilutions (10^−2^ and 10^−3^) and used to seed truncated recombinant hamster PrP (rHaPrP 90-231). Conversion curves are plotted as relative ThT fluorescence intensity (%) over time (h), which was normalized to the plateau phase intensity of untreated SMB-S15 cells. (**D**) PMCA assay assessing the replication capacity of cell lysates. Lysates from SMB-S15 cells transfected with mimics, inhibitor, or controls (NC, and the negative control for inhibitor, denoted as NC-in) were used as seeds. PMCA products were digested with PK and analyzed by Western blotting for newly generated PrP^res^. Quantitative analyses of relative PrP^res^ gray values are shown on the right. Data are presented as the mean ± SD from at least three independent experiments. For panels (**A**), (**B**), and (**D**), *n* = 3 independent experiments per group, and statistical differences were evaluated using one-way ANOVA followed by Tukey’s multiple-comparison test. For panel (**C**), RT-QuIC assays were independently performed three times. ** *p* < 0.01; NS, not significant.

To investigate the influences of miR-148a-3p on PrP^Sc^ in SMB-S15 cells, cells transfected with miR-148a-3p mimics or inhibitor were subjected to several PrP^Sc^-specific assays. PK-digested Western blotting showed that PK-resistant PrP signals were comparable between the miR-148a-3p mimics group and its corresponding control, but were slightly decreased in cells transfected with miR-148a-3p inhibitor compared with the NC-in group (Figure 4A). PrP-specific IFAs treated with 3 M Guanidine isothiocyanate (GdnSCN) revealed similar fluorescent intensities and granularities in the mimics group compared with its control, whereas the inhibitor group showed similar overall fluorescent intensity but markedly fewer PrP-positive granules than its control (Figure 4B). RT-QuIC assays using rHaPrP 90-231 as the substrate showed similar conversion curves in the mimics and control groups, with lag phases of less than 5 h. In contrast, lysates from inhibitor-treated cells showed a markedly delayed lag phase of approximately 29 h (Figure 4C). To further assess prion replication capacity, cell lysates were subjected to Protein Misfolding Cyclic Amplification (PMCA). After cyclic amplification using healthy mouse brain homogenates as the substrate, PMCA products were digested with PK and analyzed for newly generated PrP^res^. PMCA products seeded with lysates from the inhibitor-treated group exhibited markedly weaker classical three-band PrP^res^ signals compared with the corresponding NC-in control (Figure 4D). These results indicate that inhibition of miR-148a-3p partially reduces intracellular PrP^Sc^ accumulation and decreases prion seeding/replication activity in SMB-S15 cells.

### 2.5. ATP6AP2, a Predicted Target of miR-148a-3p, Is Decreased in Prion-Infected Brains and Cells

**Figure 5:**
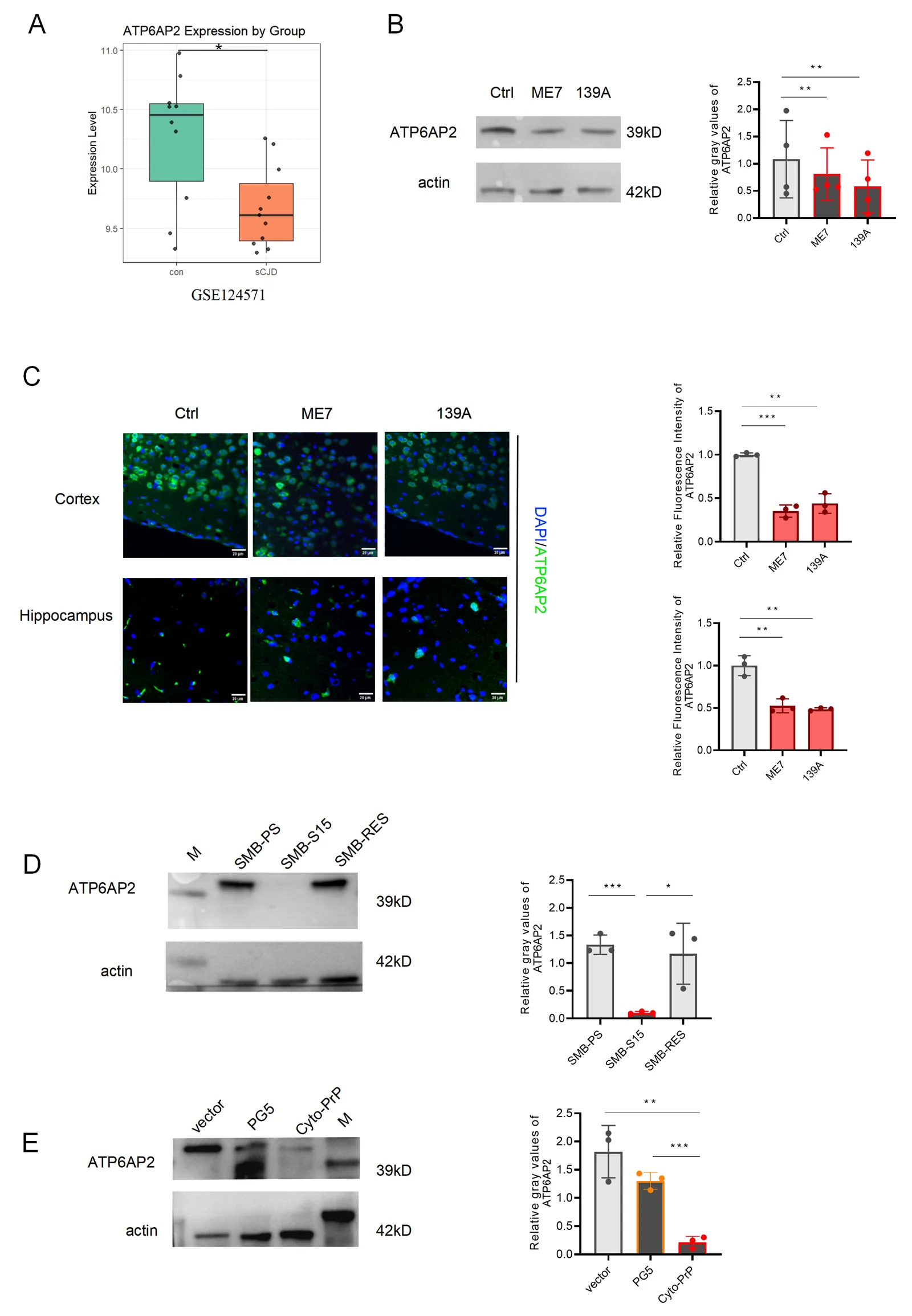
ATP6AP2 decreases in prion-infected brains and cells. (**A**) Transcriptomic analysis showing relative ATP6AP2 expression in brain tissues from sCJD patients compared to controls (con) based on the GEO dataset GSE124571. (**B**) Representative Western blot and quantitative analysis of ATP6AP2 protein levels in brain tissues from ME7- or 139A-infected mice at the terminal stage and age-matched controls (Ctrl). (**C**) Representative immunofluorescence images showing ATP6AP2 (green) distribution in the cortex and hippocampus of control (Ctrl), ME7-, and 139A-infected mice. Nuclei were counterstained with DAPI (blue). Scale bar, 200 μm. Quantitative analyses of relative ATP6AP2 fluorescence intensity in the cortex (**upper**) and hippocampus (**lower**) are shown on the right. (**D**) Western blot analysis of ATP6AP2 in the lysates of SMB-PS, SMB-S15, and SMB-RES cells. (**E**) Representative Western blot and quantitative analysis of ATP6AP2 in HEK-293T cells transiently transfected with pcDNA 3.1 vector, wild-type PrP (PG5), or cytosolic PrP mutant (Cyto-PrP). Data are presented as mean ± SD. For panel A, ATP6AP2 expression levels in the GSE124571 dataset were compared between the control and sCJD groups using a two-tailed Welch’s t-test. For panel B, *n* = 3 animals per group, and statistical differences were evaluated using one-way ANOVA followed by Tukey’s multiple-comparison test. For panel C, *n* = 3 animals per group, and one-way ANOVA followed by Tukey’s multiple-comparison test was performed separately for the cortex and hippocampus. For panels D and E, *n* = 3 independent experiments per group, and statistical differences were evaluated using one-way ANOVA followed by Tukey’s multiple-comparison test. *p < 0.05, ** *p* < 0.01, *** *p* < 0.001; NS, not significant.

Potential target genes of miR-148a-3p were further screened and scored using three databases described above. A total of 270 potential target genes were predicted. As shown in Table 1, *ATP6AP2* ranked as the top predicted target of miR-148a-3p with the highest score (0.98), whereas *PRNP* was also included among the predicted targets but with a lower score (0.66). A transcriptomic dataset of human PrDs (accession number: GSE124571) was downloaded from the Gene Expression Omnibus (GEO) database. ATP6AP2 was among the significantly downregulated differentially expressed genes (DEGs) in brain tissues from sCJD patients. Sixty-three downregulated DEGs overlapped between the miR-148a-3p predicted dataset and the human CJD dataset. Functional enrichment analysis of these 63 DEGs highlighted affected GO and KEGG pathways, including synaptic vesicle cycle and lysosomal lumen acidification, in which ATP6AP2 was included (Supplementary Figure S3). Further analysis of individual cases in the human CJD dataset revealed significantly decreased ATP6AP2 expression in sCJD patients compared with controls (Figure 5A).

To assess ATP6AP2 alterations in prion-infected mouse brains, brain samples from ME7- and 139A-infected mice at the terminal stage were analyzed. Western blotting identified markedly lower ATP6AP2 protein levels in scrapie-infected mouse brains than in control brains (Figure 5B). IFAs of brain sections showed significantly lower fluorescent intensities of ATP6AP2 signals in the regions of cortex and hippocampus of ME7- and 139A-infected mice (Figure 5C). Western blotting of three SMB cell lines revealed significantly lower ATP6AP2 levels in SMB-S15 cells than in SMB-PS and SMB-RES cells (Figure 5D). A lower ATP6AP2 level was also detected in cells expressing Cyto-PrP than in those expressing PG5-PrP (Figure 5E). These data indicate a downregulation of ATP6AP2 in the prion-infected tissues or cells.

### 2.6. Inhibition of miR-148a-3p Restores Lysosomal Acidification and Alleviates Impaired Autophagic Flux in Prion-Infected Cells by Upregulating ATP6AP2

**Figure 6:**
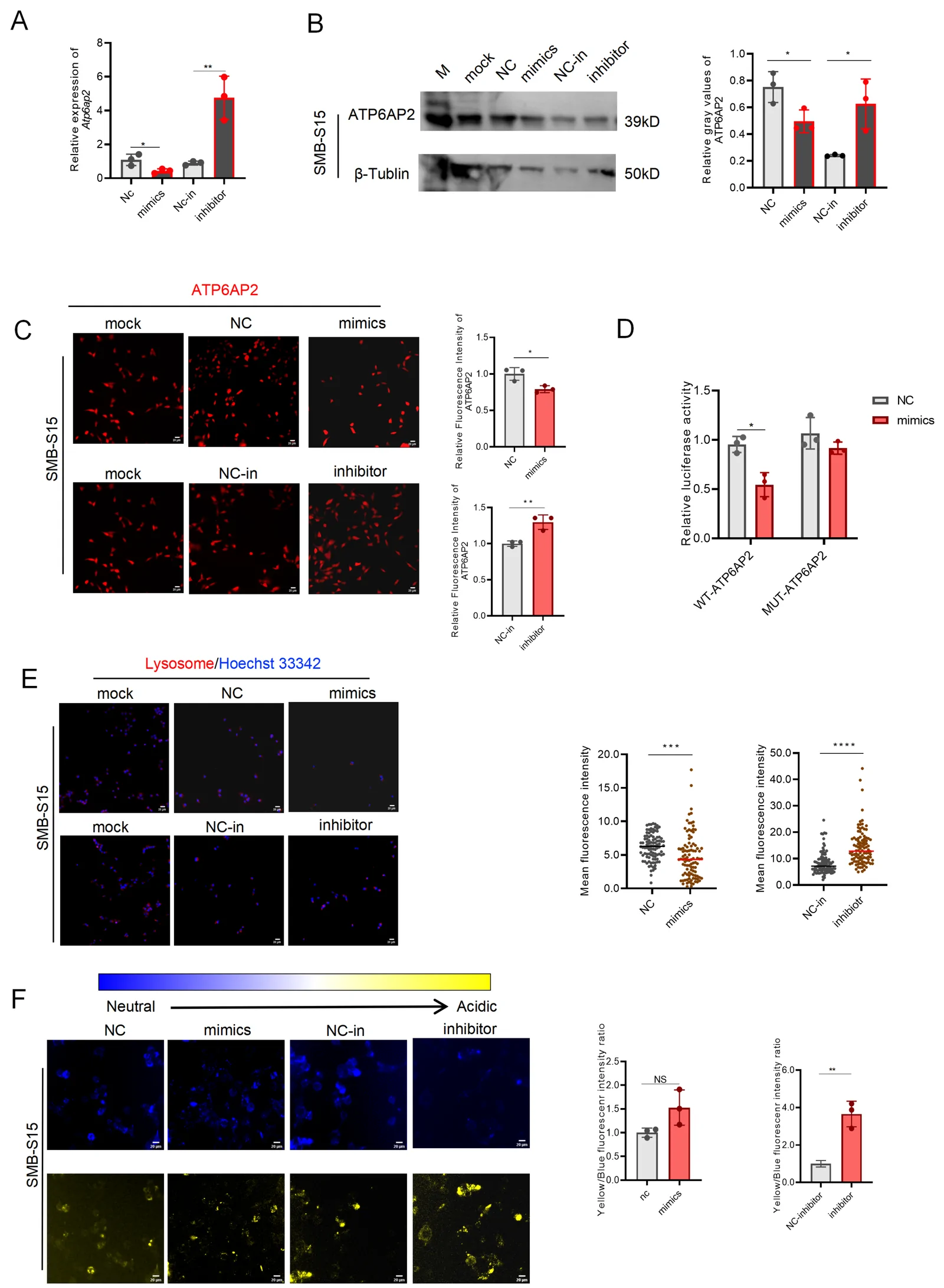

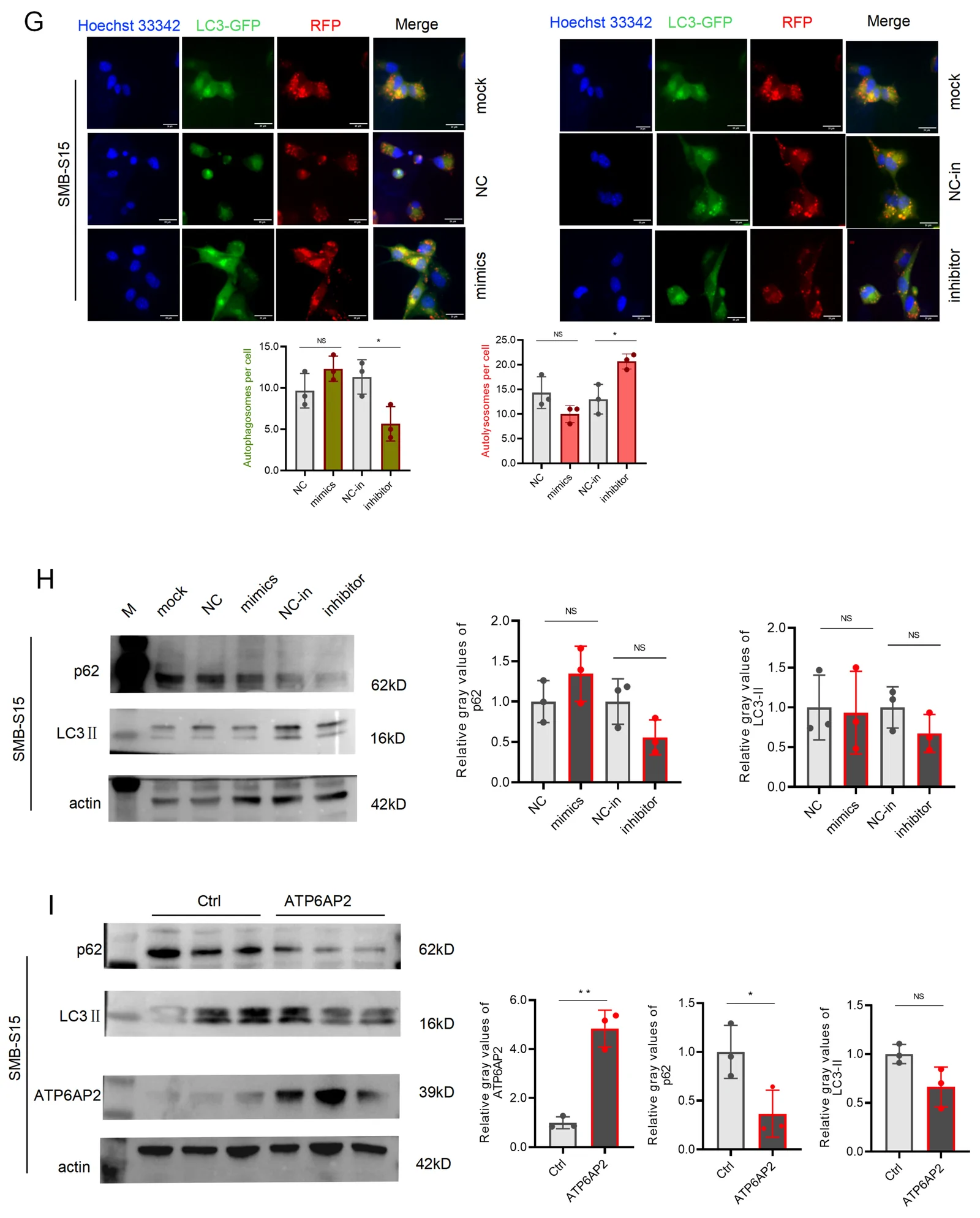

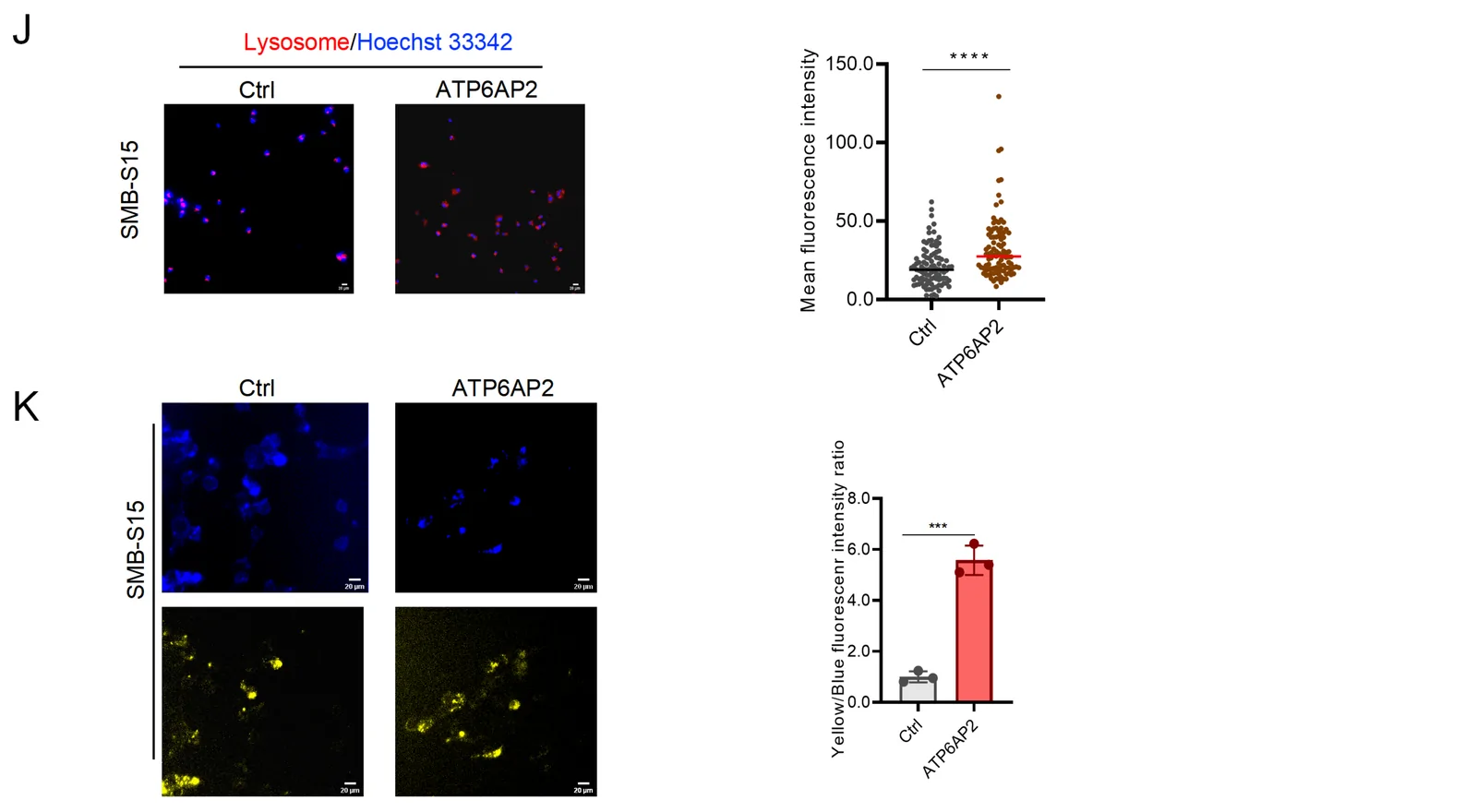
Inhibition of miR-148a-3p restores lysosomal acidification and alleviates impaired autophagic flux in prion-infected cells by upregulating ATP6AP2. (**A**–**C**) qPCR (**A**), representative Western blot (**B**) and immunofluorescence images (**C**) showing ATP6AP2 expression in SMB-S15 cells transfected with miR-148a-3p mimics, inhibitor, or their respective controls (NC, and the negative control for inhibitor, denoted as NC-in). Quantitative analyses are shown on the right. Scale bar, 20 μm. (**D**) The relative luciferase activity of HEK-293T cells co-transfected with miR-148a-3p mimics or negative control (NC) together with wt-ATP6AP2 or mut- ATP6AP2 reporter plasmids. Relative luciferase activity was calculated by normalizing firefly luciferase activity to Renilla luciferase activity. (**E**) Lysosomal acidification was measured using a lysosomal fluorescent probe. Nuclei were counterstained with Hoechst 33342 (blue). Mean fluorescence intensity per cell was quantified and plotted. Scale bar, 20 μm. For quantitative analysis, 100 cells were randomly selected from multiple fields of view across independent experiments, and the average fluorescence intensity per single cell was calculated. (**F**) Lysosomal acidification using LysoSensor™ Yellow/Blue DND-160 in SMB-S15 cells transfected with miR-148a-3p mimics or inhibitor, together with their respective negative controls (NC, NC-in). Representative blue (neutral) and yellow (acidic) fluorescence images are shown; a higher yellow/blue fluorescence intensity ratio indicates a more acidic lysosomal environment. For each independent experiment, the mean fluorescence intensities of the yellow and blue signals were measured, and the yellow/blue fluorescence intensity ratio was calculated. Scale bar, 20 μm. (**G**) Autophagic flux was monitored using the mRFP-GFP-LC3 tandem reporter. Autophagosomes are shown as yellow puncta in merged images, whereas autolysosomes are shown as red puncta. Quantitative analyses of autophagosomes and autolysosomes per cell are shown on the right. Scale bar, 20 μm. (**H**) Western blot analysis of endogenous autophagic markers p62 and LC3-II in SMB-S15 cells treated with miR-148a-3p mimics or inhibitor. (**I**) Western blot and quantitative analyses of ATP6AP2, p62, and LC3-II in SMB-S15 cells after transient overexpression of ATP6AP2 or empty vector control (Ctrl). (**J**) Lysosomal acidification assessment in SMB-S15 cells overexpressing ATP6AP2. Quantitative analysis was performed as described for panel E. (**K**) Lysosomal acidification using LysoSensor™ Yellow/Blue DND-160 in SMB-S15 cells with ATP6AP2 overexpression plasmid or empty vector control (Ctrl). Quantitative analysis was performed as described in panel F. Data are presented as the mean ± SD from at least three independent experiments. For panels (**A**–**C**), *n* = 3 independent experiments per group, and statistical differences were evaluated using one-way ANOVA followed by Tukey’s multiple-comparison test. For panel D, *n* = 3 independent experiments, and statistical differences were evaluated using two-way ANOVA followed by multiple-comparison testing. For panel E, fluorescence measurements were obtained from 100 randomly selected cells per group, and the individual cell measurements were used for statistical analysis using one-way ANOVA followed by Tukey’s multiple-comparison test. For panel F, *n* = 3 independent experiments per group, and statistical differences were evaluated using one-way ANOVA followed by Tukey’s multiple-comparison test. For panel G, *n* = 3 independent experiments per group, and statistical differences were evaluated using one-way ANOVA followed by Tukey’s multiple-comparison test. For panel H, *n* = 3 independent experiments per group, and statistical differences were evaluated using one-way ANOVA followed by Tukey’s multiple-comparison test. For panel I, *n* = 3 independent experiments, and two-tailed Student’s *t*-test was used. For panel J, fluorescence measurements were obtained from 100 randomly selected cells per group, and the individual cell measurements were used for statistical analysis using one-way ANOVA followed by Tukey’s multiple-comparison test. For panel K, n = 3 independent experiments per group, and statistical differences were evaluated using a two-tailed unpaired Student’s t-test. * *p* < 0.05, ** *p* < 0.01, *** *p* < 0.001, **** *p* < 0.0001; NS, not significant.

The influence of miR-148a-3p on the expression of ATP6AP2 in SMB-S15 cells was evaluated via transfection with miR-148a-3p mimics, inhibitor or the corresponding controls. qRT-PCR (Figure 6A) and Western blotting (Figure 6B) showed that the level of ATP6AP2 was decreased in the mimics group and increased in the inhibitor group.

IFAs consistently showed decreased ATP6AP2 signals after miR-148a-3p mimic transfection and enhanced ATP6AP2 signals after inhibitor transfection (Figure 6C), suggesting that miR-148a-3p negatively regulates the expression of ATP6AP2 in prion-infected cells. Dual-luciferase reporter assays showed that miR-148a-3p mimics significantly reduced the luciferase activity of the wild-type ATP6AP2 3′UTR reporter but not that of the seed-region mutant reporter (Figure 6D), supporting direct post-transcriptional regulation of ATP6AP2 by miR-148a-3p. Lysosomal acidification was then evaluated using the commercial lysosomal fluorescent probe LysoTracker Red DND-99. Compared to the individual controls, miR-148a-3p mimics significantly reduced lysosomal fluorescence intensity, whereas miR-148a-3p inhibitor significantly increased lysosomal fluorescence intensity (Figure 6E). Consistent results were obtained using the ratiometric probe LysoSensor Yellow/Blue DND-160: the yellow/blue fluorescence intensity ratio did not differ significantly between the mimic and NC groups but was significantly increased after inhibitor transfection (Figure 6F). Autophagic flux was monitored using the mRFP-GFP-LC3 tandem reporter. miR-148a-3p mimics tended to reduce the number of autolysosomes (red puncta), although this change did not reach statistical significance, whereas miR-148a-3p inhibitor significantly increased autolysosome formation (red puncta) and reduced autophagosome accumulation (yellow puncta) (Figure 6G).

We next assessed whether miR-148a-3p directly targets core autophagy-related genes. Target prediction was performed using multiple databases, including miRDB, TargetScan, and miRWalk, and the results are summarized in Supplementary Table S1. Database screening did not identify predicted miR-148a-3p binding sites in several major autophagy-related components, including SQSTM1 (p62), MAP1LC3B (LC3), MTOR (mTOR), BECN1 (beclin-1), and ATG5. Western blot analysis of SMB-S15 cell lysates showed a slight, non-significant increase in p62 in the mimics group and a slight, non-significant decrease in p62 and LC3-II in the inhibitor group, whereas LC3-II was essentially unchanged after mimic transfection (Figure 6H). To determine whether ATP6AP2 restoration was sufficient to improve lysosomal function, SMB-S15 cells were transfected with an ATP6AP2-expressing plasmid. CCK-8 assays showed no significant effect of ATP6AP2 plasmid transfection on cell viability at 24, 48, or 72 h (Supplementary Figure S2B). ATP6AP2 overexpression significantly reduced p62 levels and slightly, though not significantly, decreased LC3-II levels compared with the controls (Figure 6I). ATP6AP2-overexpressing cells showed significantly stronger lysosomal probe fluorescence (Figure 6J) and a significantly higher LysoSensor yellow/blue fluorescence ratio (Figure 6K), indicating improved lysosomal acidification. These results suggest that inhibition of miR-148a-3p restores lysosomal acidification and alleviates impaired autophagic flux in prion-infected cells, at least in part through upregulation of ATP6AP2.

### 2.7. Overexpression of ATP6AP2 Reduces PrP^Sc^ Accumulation and Seeding Activity in Prion-Infected Cells

**Figure 7:**
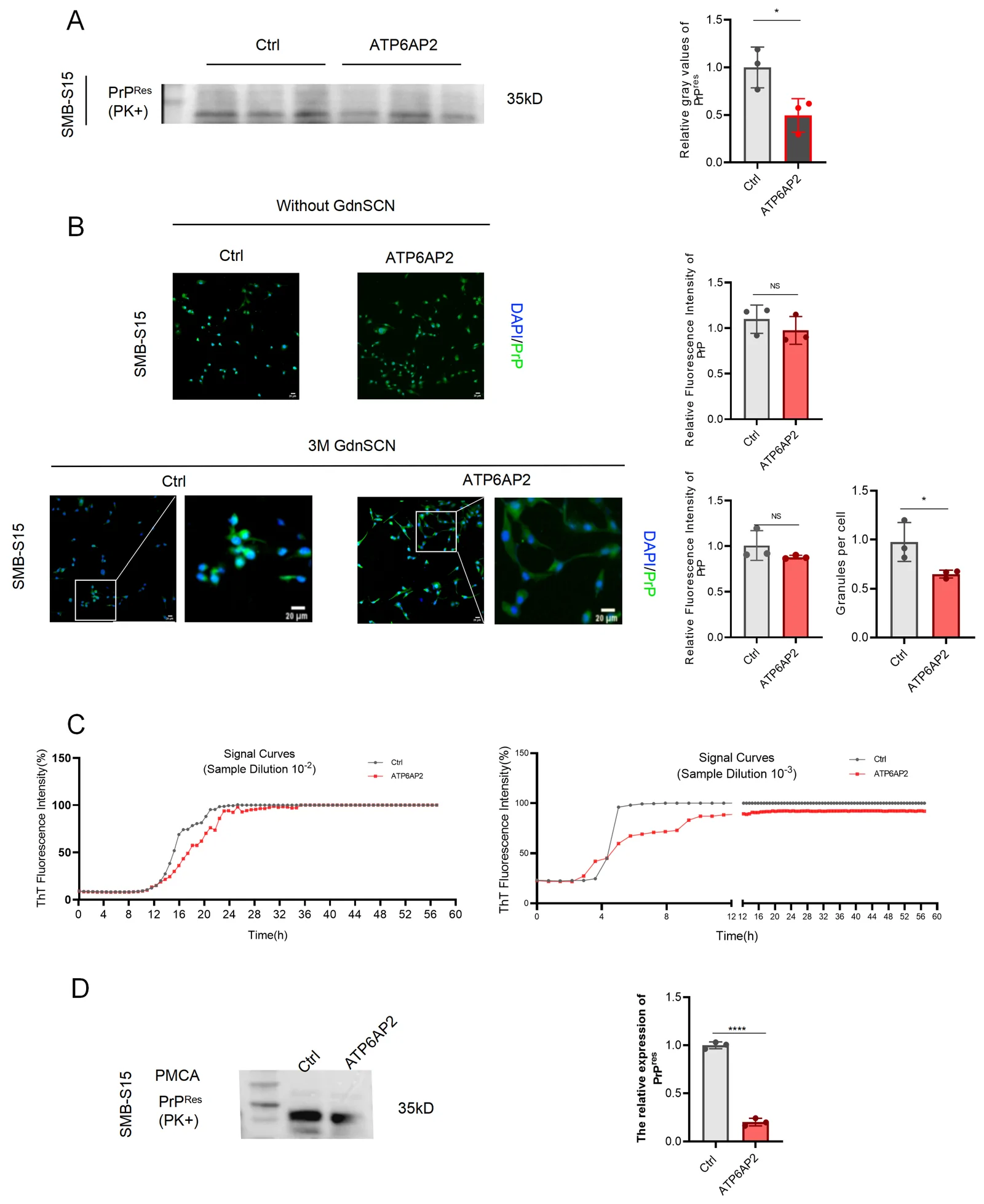
Overexpression of ATP6AP2 reduces PrP^Sc^ accumulation and seeding activity in prion-infected cells. (**A**) PK-digested Western blot analysis of protease-resistant PrP in SMB-S15 cells transiently transfected with the ATP6AP2 overexpression plasmid or empty vector control. Quantitative analysis of relative gray values of PrP^res^ is shown on the right. (**B**) Representative PrP-specific immunofluorescence images of SMB-S15 cells pre-treated with 3 M GdnSCN to expose PrP^Sc^-associated epitopes (green). Nuclei were counterstained with DAPI (blue). Quantitative analyses of relative PrP fluorescence intensity and the number of PrP-positive granules per cell are shown on the right. Scale bar, 20 μm. (**C**) RT-QuIC assay assessing the seeding activity of cell lysates from SMB-S15 cells transiently transfected with the ATP6AP2 overexpression plasmid or empty vector control. Cell lysates were serially diluted and used to seed recombinant rHaPrP 90-231 substrate. (**D**) PMCA assay assessing the replication capacity of PrP^Sc^. Lysates from ATP6AP2-overexpressing or control cells were used as seeds for PMCA. After cyclic amplification, products were digested with PK and analyzed by Western blotting for newly generated PrP^res^. Quantitative analysis of relative PrP^res^ expression is presented on the right. Data are presented as the mean ± SD from at least three independent experiments. For panels (**A**), (**B**) and (**D**), *n* = 3 independent experiments, and statistical differences were evaluated using two-tailed Student’s *t*-test. RT-QuIC assays were independently performed three times. * *p* < 0.05, **** *p* < 0.0001; NS, not significant.

To evaluate the effect of overexpression of ATP6AP2 on PrP^Sc^ in SMB-S15 cells, an ATP6AP2-expressing plasmid was transiently introduced into the cells. PK-digested Western blotting showed significantly weaker PrP^Sc^ signals after ATP6AP2 overexpression (Figure 7A), whereas total (non-PK-digested) PrP levels were not markedly altered (Supplementary Figure S4), indicating that the reduction in PK-resistant PrP was not secondary to changes in total PrP abundance. PrP-specific IFAs exposed to 3 M GdnSCN showed nearly unchanged fluorescent intensity but significantly fewer intracellular PrP-positive granules in ATP6AP2-overexpressing cells (Figure 7B). Lysates from SMB-S15 cells overexpressing ATP6AP2 and the corresponding control cells were then subjected to RT-QuIC assays. Compared with the control group, ATP6AP2 overexpression delayed the lag phase and slightly reduced the maximum ThT fluorescence intensity (Figure 7C), suggesting decreased prion seeding activity. Consistently, PMCA assays demonstrated a marked reduction in newly generated PrP^res^ signals in the ATP6AP2-overexpressing group relative to the control group (Figure 7D). These data indicate that increasing ATP6AP2 expression reduces PrP^Sc^ accumulation and seeding activity in prion-infected cells.

## 3. Discussion

PrDs are fatal neurodegenerative disorders characterized by progressive neuronal injury, spongiform degeneration, gliosis, and accumulation of misfolded PrP^Sc^. Efficient clearance of PrP^Sc^ depends on intact cellular proteostasis systems, particularly the autophagy-lysosome pathway [36,37]. In this study, we identified miR-148a-3p as an upstream regulatory factor that connects prion-associated protein accumulation with ATP6AP2-dependent lysosomal dysfunction. We show that miR-148a-3p is increased in scrapie-infected mouse brains and prion-infected SMB-S15 cells. Functionally, inhibition of miR-148a-3p restores ATP6AP2 expression, improves lysosomal acidification, alleviates impaired autophagic flux, and reduces PrP^Sc^ accumulation as well as prion seeding/replication activity in prion-infected cells.

The increase in miR-148a-3p in prion-infected models is notable because miR-148a-3p has been implicated in neuroinflammatory and neurodegenerative processes [38,39,40]. In the present study, miR-148a-3p was selectively increased in ME7- and 139A-infected mouse brains, whereas miR-148b-3p and miR-152-3p did not show comparable changes. In SMB-S15 cells, miR-148b-3p was also significantly increased, albeit to a much lesser extent than miR-148a-3p, whereas miR-152-3p remained unchanged; given the shared seed sequence within the miR-148 family, a potential contribution of miR-148b-3p to ATP6AP2 regulation cannot be fully excluded. The elevated miR-148a-3p was largely reversed in SMB-RES cells after removal of prion propagation by resveratrol, supporting an association between miR-148a-3p upregulation and the prion-infected state. In addition, increased miR-148a-3p in cells expressing cytosolic PrP suggests that abnormal PrP accumulation or mislocalization may contribute to this response. The concomitant increase in pre-miR-148a further indicates that miR-148a-3p dysregulation may occur, at least in part, at the transcriptional or processing level. The precise upstream events that drive miR-148a-3p upregulation during prion infection remain to be clarified.

A seemingly paradoxical observation is that miR-148a-3p negatively regulates PrPC expression in non-prion-infected cell lines but promotes PrP^Sc^ accumulation in prion in-fected cells. This discrepancy is likely explained by the balance between PrP^C^ substrate availability and lysosomal clearance capacity for PrP^Sc^. In non-prion-infected cells, miR-148a-3p directly targets the *PRNP* transcripts and suppresses the normal PrP expression, which undergoes a degradation pathway similar to that of other non-aggregated proteins [41,42]. However, in prion-infected cells, autophagosome–lysosome function seems to be closely associated with the clearance of aggregated PrP^Sc^ [43]. In prion-infected cells, miR-148a-3p-mediated suppression of ATP6AP2 markedly impairs autophagosome–lysosome function and weakens PrP^Sc^ clearance. It seems that the final consequence of the increased miR-148a-3p-mediated inhibition of ATP6AP2-dependent lysosomal function predominates over its downregulation of PrP^C^ as the substrate for PrP^Sc^ conversion, eventually leading to more accumulation of PrP^Sc^ represented by more PK-resistant PrP and higher seeding activity in RT-QuIC and PMCA.

ATP6AP2 appears to be a key mediator linking miR-148a-3p to lysosomal impairment in prion-infected cells. ATP6AP2 is required for V-ATPase function and lysosomal acidification, which are essential for lysosomal hydrolase maturation, autophagosome–lysosome fusion, and degradation of aggregation-prone proteins [44]. Our bioinformatic analysis ranked ATP6AP2 as the highest-scoring predicted target of miR-148a-3p. This interaction was further supported by WT and mutant ATP6AP2 3′UTR luciferase reporter assays, providing evidence that ATP6AP2 is directly regulated by miR-148a-3p. Remarkably decreased ATP6AP2 has been detected in sCJD transcriptomic data, scrapie-infected mouse brains, SMB-S15 cells, and cells expressing cytosolic PrP, accompanied by increased miR-148a-3p. Functionally, miR-148a-3p inhibition increased ATP6AP2 expression and enhanced lysosomal acidification, whereas miR-148a-3p mimics further reduced ATP6AP2 and weakened lysosomal fluorescence. Overexpression of ATP6AP2 in the cell model improved lysosomal acidification and reduced the accumulation and seeding activities of PrP^Sc^ in RT-QuIC and PMCA assays, supporting a functional role for ATP6AP2 in maintaining lysosomal homeostasis and limiting PrP^Sc^ accumulation during prion infection. Together, these findings provide evidence for a functional connection between miR-148a-3p, ATP6AP2, and PrP^Sc^ accumulation. Moreover, the reduction in PrP^Sc^ accumulation following ATP6AP2 overexpression indicates that ATP6AP2 can independently influence PrP^Sc^ levels in prion-infected cells. These findings support a model in which miR-148a-3p upregulation may contribute to PrP^Sc^ accumulation, at least in part, through suppression of ATP6AP2 and subsequent lysosomal dysfunction.

The autophagy-lysosome system has long been implicated in PrDs [45], but the mechanisms by which this protective pathway becomes insufficient during disease progression remain incompletely defined. Our data strongly suggest that miR-148a-3p-mediated ATP6AP2 suppression is one of the mechanisms contributing to this failure. To further investigate lysosomal function, we performed additional experiments using the ratiometric pH-sensitive probe LysoSensor Yellow/Blue DND-160. These measurements showed significantly enhanced lysosomal acidification following miR-148a-3p inhibition and ATP6AP2 overexpression, whereas miR-148a-3p mimic treatment showed an opposite, albeit non-significant, trend. The mRFP-GFP-LC3 reporter assays indicate that miR-148a-3p inhibition increased autolysosome formation and reduced autophagosome accumulation, suggesting an improved autophagic flux. Importantly, database screening did not identify predicted miR-148a-3p binding sites in several major autophagy-related genes, such as SQSTM1, MAP1LC3B, MTOR, BECN1, or ATG5. This finding argues against a direct targeting mechanism on these canonical autophagy genes and instead supports the interpretation that miR-148a-3p affects autophagic flux mainly through ATP6AP2-dependent lysosomal acidification. Thus, the effect of miR-148a-3p in prion-infected cells appears to occur primarily at the level of lysosomal competence rather than direct transcriptional regulation of core autophagy components. Mechanistically, ATP6AP2-associated V-ATPase function is important for maintaining the acidic lysosomal environment required for efficient degradation. Suppression of ATP6AP2 may therefore compromise lysosomal acidification and reduce the degradative capacity of lysosomes. Conversely, increased ATP6AP2 expression may improve lysosomal acidification and support the degradation of accumulated PrP^Sc^. The enhanced lysosomal acidification observed in our LysoSensor assays, together with changes in autophagic flux markers and reduced PrP^Sc^ accumulation following ATP6AP2 overexpression, is consistent with this proposed mechanism. These findings suggest that restoration of lysosomal function may contribute to the reduction in PrP^Sc^ accumulation.

From a translational perspective, our findings suggest that the miR-148a-3p/ATP6AP2 axis may represent a potential pathway for modulating lysosomal dysfunction associated with PrDs. The observation of reduced ATP6AP2 expression in the sCJD transcriptomic dataset provides preliminary evidence of the relevance of this pathway to human prion disease. Furthermore, the reduction in PrP^Sc^ accumulation and seeding activity following ATP6AP2 overexpression suggests that modulation of lysosomal function may be worth exploring as a potential intervention strategy. Nevertheless, further in vivo validation will be necessary to determine whether targeting this pathway has therapeutic potential.

Several limitations should be acknowledged. First, our findings were primarily obtained from terminal-stage brain tissues of two scrapie-infected mouse models and established cell-based prion infection systems. Whether the observed miR-148a-3p/ATP6AP2 alterations are conserved across other prion strains and different disease stages remain to be determined. Longitudinal in vivo and cell-type-specific analyses will be required to define the temporal and cellular changes in this regulatory axis during prion infection. Second, although our results support an association between miR-148a-3p/ATP6AP2 regulation, lysosomal dysfunction, and PrP^Sc^ accumulation, additional in vivo loss-of-function and rescue experiments are needed to further establish causality. The use of separate negative controls for the mimic and inhibitor experiments (NC and NC-in) may also introduce baseline differences as a potential confounding factor, although consistent trends were observed across multiple cell models. Finally, the present study focused on the basic mechanisms of the miR-148a-3p/ATP6AP2 axis rather than its therapeutic application. Therefore, its in vivo efficacy, safety, specificity, and delivery remain to be investigated. Potential off-target effects of miR-148a-3p inhibition also cannot be excluded, as miR-148a-3p has been reported to regulate other targets. Transcriptome-wide analyses, such as RNA sequencing, will be valuable for evaluating target specificity and potential off-target effects in future studies.

In summary, our study delineates a pathogenic regulatory axis in which prion-associated miR-148a-3p upregulation suppresses ATP6AP2, impairs lysosomal acidification and autophagic flux, and thereby limits PrP^Sc^ clearance. Relief of this suppression restores lysosomal homeostasis and reduces PrP^Sc^ accumulation and seeding/replication activity. These findings identify the miR-148a-3p/ATP6AP2 axis as a potential regulatory pathway and therapeutic target for PrDs.

## 4. Materials and Methods

### 4.1. Animal Models

Stored brain samples from C57BL/6 mice inoculated with the mouse-adapted scrapie strains 139A and ME7 were utilized in this study. The clinical manifestations and neuropathological features of these prion-infected mouse models have been well characterized in our previous studies [46]. The average incubation periods were 183.9 ± 23.1 days for 139A-infected mice, and 184.2 ± 11.8 days for ME7-infected mice. Age-matched mock-inoculated healthy animals were used as negative controls. Brain specimens from transgenic mouse models of other neurodegenerative diseases, including APP/PS1 mice (for AD) and A53T mice (for PD), were also included. The female animals were randomly allocated to experimental groups by age, with three animals per group, to ensure intergroup balance and minimize baseline confounding variables. All animal experiments were approved by the Animal Ethics Committee of National Institute for Viral Disease Control and Prevention, Chinese Center for Disease Control and Prevention (China CDC), under approval number 20231025083, and were conducted in strict compliance with institutional and national guidelines for the care and use of laboratory animals.

### 4.2. Cell Culture and Transfection

The scrapie Chandler strain-infected mouse cell line SMB-S15 and its uninfected counterpart (SMB-PS) were obtained from the Roslin Institute (Edinburgh, UK) [47,48]. The prion-cured cell line SMB-RES was generated by exposing SMB-S15 cells to 10 μM resveratrol for 7 days to eliminate prion replication and infectivity, as described previously [14]. Other cell lines used in this study included mouse neuroblastoma N2a cells, human neuroblastoma (SH-SY5Y, SK-N-SH) cells, and human embryonic kidney (HEK-293T) cells. All cells were maintained in Dulbecco’s modified Eagle’s medium (DMEM) supplemented with 10% fetal bovine serum (FBS) and 1% penicillin/streptomycin/amphotericin B solution in a humidified incubator with 5% CO_2_ at 37 °C. All cell lines were passaged no more than 20 times after resuscitation from frozen stocks and were routinely monitored for morphology and growth rate to minimize phenotypic drift.

The miR-148a-3p mimics, miR-148a-3p inhibitor, and their corresponding negative controls (NC and NC-inhibitor) were commercially synthesized (Beijing DIA-UP BIOTECH, Beijing, China). The sequences of these synthetic RNA oligonucleotides are listed in Supplementary Table S2. The ATP6AP2-expressing plasmid based on the pcDNA3.1(+) vector was generated by DIA-UP BIOTECH. Recombinant plasmids expressing wild-type PrP (pcDNA3.1-PrP-PG5) and cytosolic PrP (pcDNA3.1-CytoPrP) were generated previously [49]. Cells were seeded in 6- or 12-well plates and transiently transfected using Lipofectamine 3000 (Invitrogen, Thermo Fisher Scientific, Cat# L3000015, Waltham, MA, USA) according to the manufacturer’s instructions. Cells were harvested 24–48 h after transfection for subsequent analyses. For transfection in 6-well plates, RNA oligonucleotides were used at a final concentration of 40 nM, and plasmid DNA was used at 2.5 μg per well. All transfection experiments were performed in at least three independent biological replicates.

### 4.3. Dual-Luciferase Reporter Assay

All reporter plasmids were synthesized by DIA-UP BIOTECH (China) and verified by Sanger sequencing. The wild-type (WT) PRNP 3′UTR sequence encompassing the predicted miR-148a-3p binding sites was synthesized and cloned into the pmirGLO dual-luciferase reporter vector to generate the WT-PRNP reporter. The sequences of these reporter plasmids are listed in Supplementary Table S4. Wild-type (WT-ATP6AP2) and seed-region mutant (MUT-ATP6AP2) ATP6AP2 3′UTR reporter plasmids were generated using the same strategy, and the corresponding sequences are also listed in Supplementary Table S4. A mutant reporter plasmid (MUT-PRNP) was constructed by introducing site-directed mutations into the seed-matching sequences of the 3′UTR. HEK293T cells were cultured in 24-well plates and transfected with either the WT or MUT vector plasmid (0.5 μg) together with miR-148a-3p mimics (40 nM) or the corresponding negative control using Lipofectamine 3000 (Invitrogen, USA). Luciferase activity was measured 48 h after transfection using the Dual-Luciferase Reporter Assay System (Promega, Cat# E1910, Madison, WI, USA), and Renilla luciferase activity was used as an internal control. Relative luciferase activity was calculated as the ratio of firefly luciferase activity to Renilla luciferase activity.

### 4.4. Bioinformatics and Public Database Analysis

Three independent online databases miRDB, TargetScan, and miRWalk were utilized to predict potential target genes of miR-148a-3p. Candidate target genes identified by the three databases were integrated for downstream prioritization. To facilitate cross-database comparison, prediction scores from each database were normalized before calculation of an integrated weighted score. For TargetScan, the weighted context++ score was transformed so that stronger predicted repression corresponded to a higher normalized score. The final integrated score was calculated using the normalized scores from each database according to the following formula: Integrated weighted score = (normalized miRDB score × 0.4) + (normalized TargetScan score × 0.3) + (normalized miRWalk Target Score × 0.3). Predicted miR-148a-3p binding sites within the *PRNP/Prnp* and *ATP6AP2/Atp6ap2* 3′UTRs were further evaluated according to seed sequence complementarity and evolutionary conservation.

For disease-associated transcriptomic analysis, the transcriptomic dataset of human PrDs (accession numbers: GSE124571) was downloaded from the GEO database. Expression matrices were log2-transformed and quantile-normalized using the normalizeBetweenArrays function in the limma package. DEGs between CJD patients and healthy controls were identified using the limma package in R. Downregulated genes were defined as those with a nominal *p* < 0.05 and log2 Fold Change (logFC) < 0. The intersection between predicted miR-148a-3p target genes and downregulated genes was used to identify disease-relevant candidate targets. Gene Ontology Biological Process (GO-BP) and Kyoto Encyclopedia of Genes and Genomes (KEGG) enrichment analyses were performed using the clusterProfiler package, and results were visualized using the ggalluvial package together with other R packages.

### 4.5. RNA Extraction and Quantitative Real-Time PCR (qRT-PCR)

Total RNA was extracted from brain tissues or cultured cells using the RNeasy Mini Kit (QIAGEN, Cat# 74104, Hilden, Germany) and Universal miRNA MiniKit (GeneBetter, Cat# R015-50, Beijing, China) according to manufacturer’s protocol. For mRNA quantification, cDNA was synthesized using a reverse transcription kit (GeneBetter, Cat# P118, Beijing, China), and qPCR was performed using SYBR Green Master Mix (Vazyme Biotech, Cat# Q713-02, Nanjing, China) with specific primers for human PrP gene (*PRNP*) or mouse PrP gene (*Prnp*)*,* and GAPDH or mmu-Gapdh was used as the internal control for mRNA detection. For miRNA quantification, stem-loop reverse transcription primers were used, and the expression levels of miR-148a-3p, miR-148b-3p, and miR-152-3p were normalized to U6 snRNA. The pre-miR-148a related sequences were designed and synthesized by RiboBio (Guangzhou RiboBio, Guangzhou, China). All other primer sequences are provided in Supplementary Table S3. PCR amplification was performed in triplicate under the following conditions: 95 °C for 30 s, followed by 40 cycles of 95 °C for 30 s, 60 °C for 30 s, and 72 °C for 60 s. Relative expression levels were calculated using the 2^−ΔΔCt^ method.

### 4.6. Preparation of Brain and Cellular Lysates

Brain homogenates or cellular lysates (10%, *w*/*v*) were prepared as described previously [50]. Briefly, whole brain tissues or harvested cultured cells were homogenized in ice-cold lysis buffer (100 mM NaCl, 10 mM EDTA, 0.5% Nonidet P-40, 0.5% sodium deoxycholate, 10 mM Tris-HCl, pH 7.5) with protease inhibitor cocktail set III (Merck, Cat# 535140, Darmstadt, Germany). Tissue debris was removed by low-speed centrifugation at 2000× *g* for 10 min at 4 °C, and the supernatants were collected for subsequent analyses.

### 4.7. Western Blotting

Aliquots of brain homogenates or cellular lysates were separated by sodium dodecyl sulfate–polyacrylamide gel electrophoresis (SDS-PAGE) using 4–20% FuturePAGE™ Bis-Tris precast gels (ACE, Changzhou, Cat# ET10420LGel, China) in MOPS-SDS running buffer at a constant voltage of 120 V for approximately 60 min. Proteins were then transferred onto 0.45 μm PVDF membranes (Millipore, Cat# IPVH00010, Billerica, Massachusetts, USA). After blocking with QuickBlock™ blocking buffer (Beyotime Biotechnology, Cat# P0270, Shanghai, China), membranes were incubated overnight at 4 °C with primary antibodies against PrP (1:5000, Abcam, Cat# ab52604, Cambridge, UK), ATP6AP2/Renin Receptor (1:5000, Immunoway, Cat# YT4048, San Jose, CA, USA), p62 (1:2000, Immunoway, Cat#YS0004, San Jose, CA, USA), LC3B (1:5000, Abmart, China, Cat# T55992S), β-tubulin (1:10,000, Immunoway, Cat# YM960751, San Jose, CA, USA) and β-actin (1:4000, ZSGB-BIO, Cat# TA-09, Beijing, China), respectively. After incubation with HRP-conjugated secondary antibodies (1:10,000, Jackson, Cat# 115-035-003 / Cat# 111-035-003, ImmunoResearch Laboratories, West Grove, PA, USA), protein bands were visualized using an enhanced chemiluminescence (ECL) system and quantified using ImageJ software (v1.52). For detection of protease-resistant PrP, lysates were treated with proteinase K (PK, 20 μg/ml) at 37 °C for 1 h prior to SDS-PAGE.

### 4.8. Immunofluorescence Assay (IFA)

Cells grown on 4- or 8-well glass slides (Merck Millipore, Cat# PEZGS0416, Billerica, Massachusetts, USA) were fixed with 4% paraformaldehyde for 20 min, permeabilized with 0.25% Triton X-100 in phosphate-buffered saline (PBS) for 20 min at 4 °C, and blocked with 10% normal goat serum for 1 h. Brain tissues from normal, 139A-infected or ME7-infected mice were fixed in 10% buffered formalin, and paraffin sections with a thickness of 5 μm were prepared routinely. Brain sections were permeabilized with 0.3% Triton X-100 in PBS for 30 min and blocked with normal goat serum for 1 h. Cells or tissue sections were incubated with primary antibodies against PrP (1:200) or ATP6AP2 (1:300) at 4 °C overnight, followed by incubation with secondary antibodies (1:200, Alexa Fluor 488 or 594, Invitrogen, Thermo Fisher Scientific, Cat# A21210/A11062, Waltham, MA, USA) for 1 h in the dark. Nuclei were counterstained with DAPI. For PrP^Sc^-associated immunofluorescence, cells were treated with 3 M GdnSCN for 4 min prior to the routine immunostaining procedure. Images were captured using an ImageXpress Micro Confocal system (Molecular Devices, Shanghai, China) and analyzed using ImageJ software.

### 4.9. Lysosomal Acidification and Autophagic Flux Assessment

Lysosomal acidification was evaluated using a lysosomal fluorescent probe, including LysoTracker Red DND-99 (Thermo Fisher Scientific, Cat#L12492, Eugene, OR, USA). Cells were incubated with the probe at 37 °C for 30 min, washed twice with PBS, and immediately imaged. For quantitative analysis, cells were randomly selected from multiple fields of view across independent experiments, and the mean lysosomal fluorescence intensity per cell was quantified from randomly selected microscopic fields using ImageJ software.

In addition, lysosomal acidification was also measured using LysoSensor Yellow/Blue DND-160 (Thermo Fisher Scientific, Cat# L7545, Eugene, OR, USA). Cells were incubated with the probe (1μmol/L) at 37 °C for 10–15 min, washed with PBS, and immediately imaged. Blue and yellow fluorescence images were acquired under identical imaging settings for all experimental groups. The fluorescence intensity ratio of yellow to blue was calculated for each sample and used as a relative indicator of lysosomal acidification [51]. LysoSensor Yellow/Blue DND-160 is a ratiometric fluorescent probe whose excitation and emission properties are pH dependent. The blue fluorescence signal is primarily detected at approximately 329 nm excitation/440 nm emission, whereas the yellow fluorescence signal is associated with approximately 384 nm excitation/540 nm emission under acidic conditions. For each independent experiment, three replicate experiments were performed, and multiple randomly selected fields were imaged for each group. The mean fluorescence intensity of the blue and yellow channels was measured for each field under identical image-analysis conditions. The relative lysosomal acidification was evaluated based on the ratio of yellow to blue fluorescence intensity, with a higher yellow/blue ratio indicating relatively increased lysosomal acidity.

Autophagic flux was monitored using the mRFP-GFP-LC3 tandem reporter plasmid (HanBio Biotechnology, Shanghai, China). Because GFP fluorescence is quenched in the acidic lysosomal environment whereas RFP fluorescence remains relatively stable, autophagosomes were identified as yellow puncta (GFP^+^/RFP^+^) in merged images, while autolysosomes were identified as red puncta (GFP^−^/RFP^+^). Autophagic flux was assessed by quantifying the average numbers of yellow and red LC3 puncta per cell.

### 4.10. Real-Time Quaking-Induced Conversion (RT-QuIC) Assay

The seeding activity of PrP^Sc^ was assessed using an RT-QuIC assay as described previously [52,53,54]. Reactions were performed in black 96-well plates with clear bottoms, with a final volume of 100 μL per well. Each reaction contained 0.1 mg/ml purified recombinant truncated hamster PrP (rHaPrP 90-231) as the substrate, 10 μM Thioflavin T (ThT), 170 mM NaCl, 1 mM EDTA, 1× PBS (pH 7.4), and 1 µL of 10^−2^ and 10^−3^ diluted cell lysate. Each sample was tested in quadruplicate. Plates were sealed and incubated in a FLUOstar Omega microplate reader (BMG LABTECH GmbH, Ortenberg, Germany) at 42 °C. The standard protocol consisted of alternating cycles of shaking (700 rpm, double orbital, 60 s) and resting (60 s) for 60–90 h. ThT fluorescence was measured every 45 min using excitation at 450 nm and emission at 480 nm. Lag phase and maximum fluorescence intensity were calculated to evaluate prion seeding activity.

### 4.11. Protein Misfolding Cyclic Amplification (PMCA)

PMCA was conducted as described previously using a Misonix sonicator 3000/4000 (Misonix, Farmingdale, NY, USA) containing an adjustable temperature water bath circulation system and a microplate horn for PCR tubes [55]. PMCA reactions contained 10 μL of 10^−3^ diluted SMB-S15 cell lysates as the seed and 90 μL of substrate (10% healthy mouse brain homogenate). Thin-walled 0.2 mL PCR tubes containing 100 μL reaction mixtures were placed in a floating position in the sonicator. Each amplification cycle consisted of 20 s sonication (power setting 9.5) followed by incubation at 37 °C for 29 min and 40 s. A total of 81 cycles were performed for each reaction. PMCA products were subsequently digested with PK and analyzed by Western blotting to detect newly generated PrP^res^.

### 4.12. Statistical Analysis

All data were analyzed using GraphPad Prism (version 8.0) and are presented as the mean ± standard deviation (SD) from at least three independent experiments unless otherwise indicated. Comparisons between two groups were performed using a two-tailed Student’s *t*-test, whereas comparisons among multiple groups were analyzed using one-way ANOVA followed by Tukey’s multiple-comparison test. A *p*-value of < 0.05 was considered statistically significant. Statistical significance is indicated as follows: * *p* < 0.05, ** *p* <0.01, *** *p* <0.001, **** *p* <0.0001.

## Figures and Tables

**Table 1:** Predicted target genes of miR-148a-3p from the miRDB, TargetScan and miRWalk databases.

	miRNA ID	Symbol Gene	Weighted Score	Protein
1	miR-148a-3p	ATP6AP2	0.98	ATPase H^+^ transporting accessory protein 2
2	miR-148a-3p	NPTN	0.84	Neuroplastin
3	miR-148a-3p	QKI	0.80	QKI, RNA binding effector protein
4	miR-148a-3p	B4GALT5	0.79	Beta-1,4-galactosyltransferase 5
5	miR-148a-3p	WDR47	0.79	WD repeat domain 47
6	miR-148a-3p	TGFA	0.76	Transforming growth factor alpha
7	miR-148a-3p	FXR1	0.74	Fragile X mental retardation, autosomal homolog 1
8	miR-148a-3p	C18ORF25	0.74	Chromosome 18 open reading frame 25
9	miR-148a-3p	CLCN6	0.73	Chloride voltage-gated channel 6
10	miR-148a-3p	MAP3K9	0.73	Mitogen-activated protein kinase 9
11	miR-148a-3p	B4GALT6	0.73	Beta-1,4-galactosyltransferase 6
12	miR-148a-3p	MITF	0.71	Melanocyte-inducing transcription factor
13	miR-148a-3p	ERBB3	0.71	Erb-B2 receptor tyrosine kinase 3
14	miR-148a-3p	DCP2	0.71	Decapping mRNA 2
15	miR-148a-3p	MMP15	0.71	Matrix metallopeptidase 15
16	miR-148a-3p	LRP2	0.71	LDL receptor related protein 2
17	miR-148a-3p	INO80	0.71	INO80 complex subunit
18	miR-148a-3p	XPO4	0.71	Exportin 4
19	miR-148a-3p	CCT6A	0.71	Chaperonin containing TCP1 subunit 6A
20	miR-148a-3p	ESR1	0.70	Estrogen receptor 1
21	miR-148a-3p	ARRDC3	0.68	Arrestin domain containing 3
22	miR-148a-3p	TMEM170A	0.68	Transmembrane protein 170A
23	miR-148a-3p	SRSF11	0.68	Serine and arginine rich splicing factor 11
24	miR-148a-3p	LBR	0.67	Lamin B receptor
25	miR-148a-3p	KAT7	0.67	Lysine acetyltransferase 7
26	miR-148a-3p	DOCK6	0.67	Dedicator of cytokinesis 6
27	miR-148a-3p	WDR20	0.67	WD repeat domain 20
28	miR-148a-3p	LMTK2	0.67	Lemur tyrosine kinase 2
29	miR-148a-3p	USP48	0.67	Ubiquitin specific peptidase 48
30	miR-148a-3p	AGO4	0.67	Argonaute RISC component 4
31	miR-148a-3p	RPS6KA5	0.66	Ribosomal protein S6 kinase A5
32	miR-148a-3p	PRNP	0.66	Prion protein
33	miR-148a-3p	ELAVL4	0.65	ELAV-like protein 4
34	miR-148a-3p	BMP3	0.65	Bone morphogenetic protein 3
35	miR-148a-3p	FBN1	0.64	Fibrillin-1
36	miR-148a-3p	FMR1	0.64	Fragile X messenger ribonucleoprotein 1
37	miR-148a-3p	CNTN4	0.64	Contactin-4
38	miR-148a-3p	CS	0.64	Citrate synthase
39	miR-148a-3p	TEK	0.64	Tyrosine-protein kinase receptor TEK
40	miR-148a-3p	RASSF8	0.64	Ras association domain-containing protein 8
41	miR-148a-3p	CUL5	0.64	Cullin-5
42	miR-148a-3p	CBLB	0.63	E3 ubiquitin-protein ligase CBL-B
43	miR-148a-3p	CDKN1B	0.63	Cyclin-dependent kinase inhibitor 1B
44	miR-148a-3p	OSBPL11	0.63	Oxysterol-binding protein-related protein 11
45	miR-148a-3p	EYA3	0.63	Eyes absent homolog 3
46	miR-148a-3p	LYSMD2	0.63	LysM and putative peptidoglycan-binding domain-containing protein 2
47	miR-148a-3p	ITSN2	0.63	Intersectin-2
48	miR-148a-3p	LEPROTL1	0.63	Leptin receptor overlapping transcript-like 1
49	miR-148a-3p	GTF2H1	0.63	General transcription factor IIH subunit 1
50	miR-148a-3p	CAND1	0.63	Cullin-associated NEDD8-dissociated protein 1

## Data Availability

The original contributions presented in this study are included in the article. Further inquiries can be directed to the corresponding authors.

## Supplementary Materials

The following supporting information can be downloaded at: https://www.mdpi.com/article/10.3390/ijms27219524/s1.

## Abbreviations

The following abbreviations are used in this manuscript:

ATP6AP2	ATPase H+ transporting accessory protein 2
AD	Alzheimer’s disease
BSE	Bovine spongiform encephalopathy
CJD	Creutzfeldt–Jakob Disease
CNS	Central nervous system
CSF	Cerebrospinal fluid
CWD	Chronic wasting disease
DEGs	Differentially expressed genes
DMEM	Dulbecco’s modified Eagle’s medium
ECL	Enhanced chemiluminescence
FBS	Fetal bovine serum
GdnSCN	Guanidine isothiocyanate
GEO	Gene Expression Omnibus
GO-BP	Gene Ontology Biological Process
GPI	Glycosylphosphatidylinositol
KEGG	Kyoto Encyclopedia of Genes and Genomes
LogFC	Log_2_ Fold Change
miRNA	MicroRNA
PBS	Phosphate-buffered saline
PD	Parkinson’s disease
PMCA	Protein Misfolding Cyclic Amplification
PK	Proteinase K
PrDs	Prion diseases
pre-miR-148a	Precursor of miR-148a
PrP^C^	Cellular prion protein
PrP^res^	Proteinase K (PK)-resistant PrP
PrP^Sc^	Scrapie prion protein
qRT-PCR	Quantitative real-time PCR
RISC	RNA-induced silencing complex
RT-QuIC	Real-time quaking-induced conversion
sCJD	Sporadic Creutzfeldt–Jakob disease
SD	Standard deviation
SPF	Specific pathogen-free
ThT	Thioflavin T
TSE	Transmissible spongiform encephalopathy
V-ATPase	Vacuolar-type H+-ATPase
WB	Western blot
XPDS	X-linked parkinsonism with spasticity
