# Supplementary material for: Inhibition of miR-148a-3p Ameliorates Prion Accumulation by Restoring ATP6AP2-Mediated Lysosomal Acidification
Source: Int J Mol Sci. 2026 Oct 24;27(21):9524. doi: 10.3390/ijms27219524 (PMC13762750; doi:10.3390/ijms27219524)
Supplement: Supplementary file 1 [file ijms-27-09524-s001.zip › ijms-4525770-supplementary.pdf]

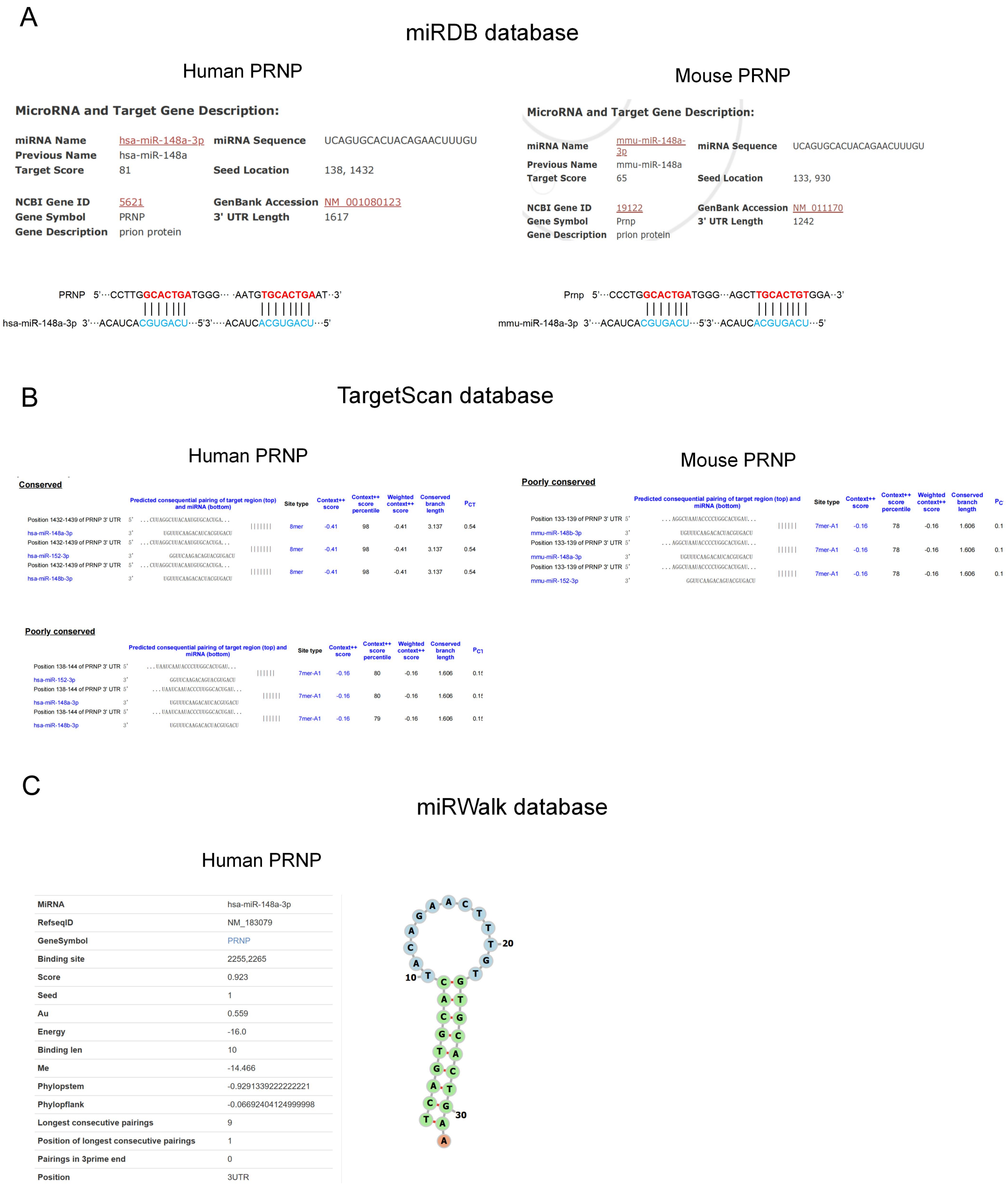

Supplementary Fig. 1

Supplementary Fig. 1 Raw prediction data from online bioinformatic databases indicating *PRNP* as a target of miR-148a-3p. Representative screenshots show the predicted binding parameters, target scores, and sequence alignments for miR-148a-3p and *PRNP/Prnp* from the miRDB (A), TargetScan (B), and miRWalk (C), respectively.

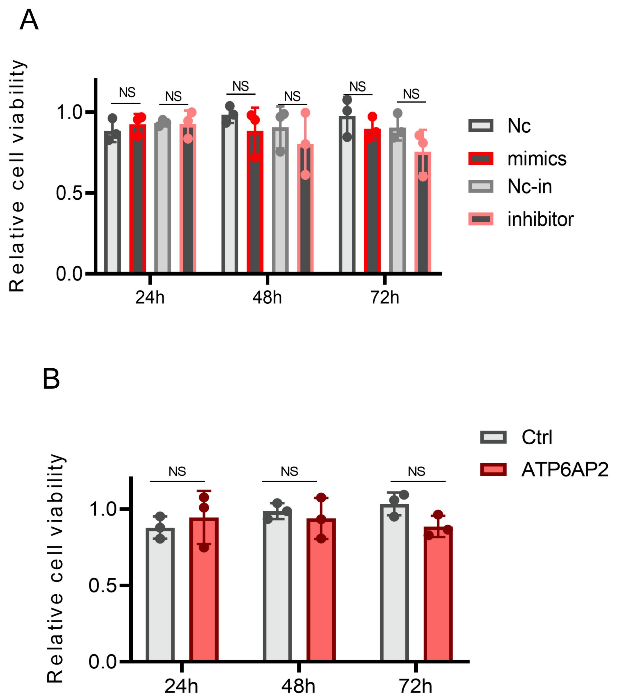

Supplementary Fig. 2

Supplementary Fig. 2 Cell viability assessed by CCK-8 assay. **A** SMB-S15 cells transfected with miR-148a-3p mimics, inhibitor, or their respective negative controls (NC, and the negative control for inhibitor, denoted as NC-in) were assayed at 24, 48, and 72 h post-transfection. B. SMB-S15 cells transiently transfected with the ATP6AP2-expressing plasmid or empty vector control (Ctrl) were assayed at 24, 48, and 72 h post-transfection. Relative cell viability was

normalized to the corresponding control group at each time point. Data are presented as the mean ± SD from three independent experiments (n = 3). Statistical analysis was performed using two-way ANOVA followed by multiple-comparison testing for comparisons between the indicated groups at each time point. NS, not significant.

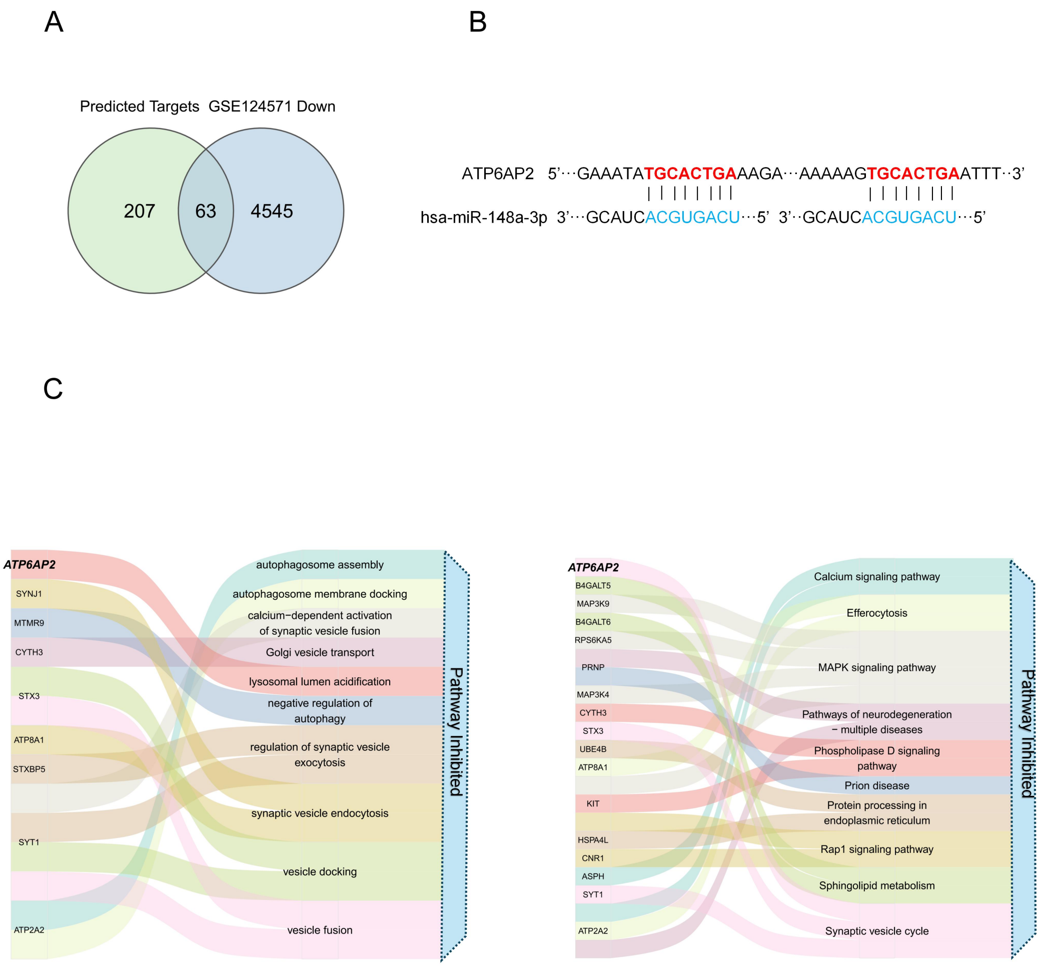

Supplementary Fig. 3

Supplementary Fig. 3 Bioinformatic screening identifies ATP6AP2 as a candidate miR-148a-3p target associated with lysosomal acidification. **A** Venn diagram showing the intersection between the 270 predicted target genes of miR-148a-3p derived from miRDB, TargetScan, and miRWalk and the significantly DEGs identified in the sCJD transcriptomic dataset GSE124571. **B** Schematic sequence alignment demonstrating conserved putative binding sites for the miR-148a-3p seed sequence within the 3' UTR of ATP6AP2. The complementary seed-matching sequences are highlighted in red and blue. **C** Functional enrichment and pathway mapping of overlapping candidate genes, highlighting pathways related to lysosomal lumen acidification and other disease-relevant processes. Data were generated from bioinformatic prediction and public transcriptomic dataset analysis.

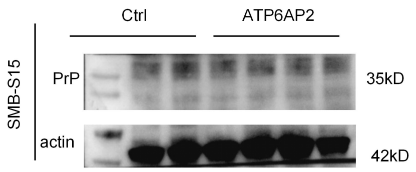

Supplementary Fig. 4

Supplementary Fig. 4. Representative Western blot of total (non-PK-digested) PrP and actin levels in SMB-S15 cells transiently transfected with an ATP6AP2-expressing plasmid or empty vector control (Ctrl), showing that ATP6AP2 overexpression does not markedly alter total cellular PrP abundance.

Supplementary Table 1. Predicted targeting scores of miR-148a-3p for core autophagy components across multiple databases

Gene Symbol	miRDB Target Score	TargetScan Site type	TargetScan Weighted context++ score	miRWalk Target Score	Biological Function
ATP6AP2	97	8mer	-0.67	1	Lysosomal acidification / V-ATPase assembly
SQSTM1 (p62)	N.D.	None	None	None	Autophagy receptor
MAP1LC3B (LC3)	N.D.	None	None	None	Autophagosome elongation and closure
MTOR（mTOR）	N.D.	None	None	None	Autophagy core inhibitory kinase
BECN1 (Beclin-1)	N.D.	None	None	None	Key autophagy initiation complex
ATG5	N.D.	None	None	None	Autophagosome membrane elongation

Supplementary Table 2 Synthetic RNA oligonucleotides

RNA oligonucleotides	Sequence(5’-3’)
NC	UUGUACUACACAAAAGUACUG
	GUACUUUUGUGUAGUACAAUU
mimics	UCAGUGCACUACAGAACUUUGU
	AAAGUUCUGUAGUGCACUGAUU
NC-in	(mC)(mA)(mG)(mU)(mA)(mC)(mU)(mU)(mU)(mU)(mG)(mU)(mG)(mU)(mA)(mG)(mU)(mA)(mC)(mA)(mA)
inhibitor	(mA)(mC)(mA)(mA)(mA)(mG)(mU)(mU)(mC)(mU)(mG)(mU)(mA)(mG)(mU)(mG)(mC)(mA)(mC)(mU)(mG)(mA)

Supplementary Table 3 Primer sequences used for Reverse Transcription (RT) and quantitative Real-Time PCR (qRT-PCR).

Gene / miRNA	Type	Sequence(5'-3')
miR-148a-3p	Forward	GCGCGTCAGTGCACTACAGAA
	Reverse	ATCCAGTGCAGGGTCCGAGG
	RT	GTCGTATCCAGTGCAGGGTCCGAGGTATTCGCACTGGATACGACACAAAG
mmu-miR-148b-3p	Forward	CGCGTCAGTGCATCACAGAA
	Reverse	AGTGCAGGGTCCGAGGTATT
	RT	GTCGTATCCAGTGCAGGGTCCGAGGTATTCGCACTGGATACGACACAAAG
mmu-miR-152-3p	Forward	CGCGTCAGTGCATGACAGA
	Reverse	AGTGCAGGGTCCGAGGTATT
	RT	GTCGTATCCAGTGCAGGGTCCGAGGTATTCGCACTGGATACGACCCAAGT
U6	Forward	CTTCGGCAGCACATATACTA
	Reverse	TATGGAACGCTTCACGAATT

Gene / miRNA	Type	Sequence(5’-3’)
	RT	AAAATATGGAACGCTTCACGAATTTG
	Forward	CTCGCTTCGGCAGCACA
U6	Reverse	AACGCTTCACGAATTTGCGT
	RT	CTGCGCAAGGATGACACGC
PRNP	Forward	ACAACTTTGTGCACGACTGC
	Reverse	GGTGAAGTTCTCCCCCTTGG
Prnp	Forward	GGAGAACTTCACCGAGACCG
	Reverse	TAGGCCTGGGACTCCTTCTG
GAPDH	Forward	TCCTGGTATGACAACGAAT
	Reverse	GGTCTCTCTCTTCCTCTTG
Gapdh	Forward	AGGTCGGTGTGAACGGATTTG
	Reverse	TGTAGACCATGTAGTTGAGGTCA

Supplementary Table 4 Sequences of the wild-type and mutant 3'UTR reporters.

Reporter	Sequence
WT-PRNP	CTCGAGGGGAGGCCTTCCTGCTTGTTCCTTCGCATTCTCGTGGTCTAGGCTGGGGGAGGGGTTATCCACCTGTAGCTCTTTCAAT<br>TGAGGTGGTTCTCATTCTTGCTTCTCTGTGTCCCCCATAGGCTAATACCCCTGGCACTGATGGGCCCTGGGAAATGTACAGTAGA<br>CCAGTTGCTCTTTGCTTCAGGTCCCTTTGATGGAGTCTGTCATCAGCCAGTGCTAACACCGGGCCAATAAGAATATAACACCAAAT<br>AACTGCTGGCTAGTTGGGGCTTTGTTTTGGTCTAGTGAATAAATACTGGTGTATCCCCTGACTTGTACCCAGAGTACAAGGTGACA<br>GTGACACATGTAACTTAGCATAGGCAAAGGGTTCTACAACCAAAGAAGCCACTGTTTGGGGATGGCGCCCTGGAAAACAGCCTC<br>CCACCTGGGATAGCTAGAGCATCCACACGTGGAATTCTTTCTTTACTAACAAACGATAGCTGATTGAAGGCAACAGGAAAAAAAAA<br>ATCAAATTGTCCTACTGACGTTGAAAGCAAACCTTTGTTCATTCCCAGGGCACTAGAATGATCTTTAGCCTTGCTTGGATTGAACTA<br>GGAGATCTTGACTCTGAGGAGAGCCAGCCCTGTAAAAAGCTTGGTCCTCCTGTGACGGGAGGGATGGTTAAGGTACAAAGGCTA<br>GAAACTTGAGTTTCTTCATTTCTGTCTCACAATTATCAAAAGCTAGAATTAGCTTCTGCCCTATGTTTCTGTACTTCTATTTGAACTG<br>GATAACAGAGAGACAATCTAAACATTCTCTTAGGCTGCAGATAAGAGAAGTAGGCTCCATTCCAAAGTGGGAAAGAAATTCTGCTA<br>GCATTGTTTAAATCAGGCAAAATTTGTTCCTGAAGTTGCTTTTTACCCCAGCAGACATAAACTGCGATAGCTTCAGCTTGCACTGT<br>GGATTTTCTGTATAGAATATATAAAACATAACTTCAAGCTTATGTCTTCTTTTTAAAACATCTGAAGTATGGGACGCCCTGGCCGTT<br>CCATCCAGTACTAAATGCTTACCGTGTGACCCTTGGGCTTTCAGCGTGCACTCAGTTCCGTAGGATTCCAAAGCAGACCCCTAGC<br>TGGTCTTTGAATCTGCATGTACTTCACGTTTTCTATATTTGTAACTTTGCATGTATTTTGTTTTGTCATATAAAAAGTTTATAAATGTT<br>TGCTATCAGACTGACATTAAATAGAAGCTATGATGAACACCTGGGTCGAC
MUT-PRNP	CTCGAGGGGAGGCCTTCCTGCTTGTTCCTTCGCATTCTCGTGGTCTAGGCTGGGGGAGGGGTTATCCACCTGTAGCTCTTTCAAT<br>TGAGGTGGTTCTCATTCTTGCTTCTCTGTGTCCCCCATAGGCTAATACCCCTGCGTGACTTGGGCCCTGGGAAATGTACAGTAGA<br>CCAGTTGCTCTTTGCTTCAGGTCCCTTTGATGGAGTCTGTCATCAGCCAGTGCTAACACCGGGCCAATAAGAATATAACACCAAAT<br>AACTGCTGGCTAGTTGGGGCTTTGTTTTGGTCTAGTGAATAAATACTGGTGTATCCCCTGACTTGTACCCAGAGTACAAGGTGACA<br>GTGACACATGTAACTTAGCATAGGCAAAGGGTTCTACAACCAAAGAAGCCACTGTTTGGGGATGGCGCCCTGGAAAACAGCCTC<br>CCACCTGGGATAGCTAGAGCATCCACACGTGGAATTCTTTCTTTACTAACAAACGATAGCTGATTGAAGGCAACAGGAAAAAAAAA<br>ATCAAATTGTCCTACTGACGTTGAAAGCAAACCTTTGTTCATTCCCAGGGCACTAGAATGATCTTTAGCCTTGCTTGGATTGAACTA

	GGAGATCTTGACTCTGAGGAGAGCCAGCCCTGTAAAAAGCTTGGTCCTCCTGTGACGGGAGGGATGGTTAAGGTACAAAGGCTA<br>GAAACTTGAGTTTCTTCATTTCTGTCTCACAATTATCAAAAGCTAGAATTAGCTTCTGCCCTATGTTTCTGTACTTCTATTTGAACTG<br>GATAACAGAGAGACAATCTAAACATTCTCTTAGGCTGCAGATAAGAGAAGTAGGCTCCATTCCAAAGTGGGAAAGAAATTCTGCTA<br>GCATTGTTTAAATCAGGCAAAATTTGTTCCTGAAGTTGCTTTTTACCCCAGCAGACATAAACTGCGATAGCTTCAGCTACGTGACT<br>GGATTTTCTGTATAGAATATATAAAACATAACTTCAAGCTTATGTCTTCTTTTTAAAACATCTGAAGTATGGGACGCCCTGGCCGTT<br>CCATCCAGTACTAAATGCTTACCGTGTGACCCTTGGGCTTTCAGCGTGCACTCAGTTCCGTAGGATTCCAAAGCAGACCCCTAGC<br>TGGTCTTTGAATCTGCATGTACTTCACGTTTTCTATATTTGTAACTTTGCATGTATTTTGTTTTGTCATATAAAAAGTTTATAAATGTT<br>TGCTATCAGACTGACATTAAATAGAAGCTATGATGAACACCTGGGTCGAC
WT-ATP6AP2	ATGTTACCTGTGCCAGAATTAGAAAAGGGGGTTGGAAATTGGCTGTTTTGTTAAAATATATCTTTTAGTGTGCTTTAAAGTAGATAG<br>TATACTTTACATTTATAAAAAAAAATCAAATTTTGTTCTTTATTTTGTGTGTGCCTGTGATGTTTTTCTAGAGTGAATTATAGTATTGA<br>CGTGAATCCCACTGTGGTATAGATTCCATAATATGCTTGAATATTATGATATAGCCATTTAATAACATTGATTTCATTCTGTTTAATG<br>AATTTGGAAATATGCACTGAAAGAAATGTAAAACATTTAGAATAGCTCGTGTTATGGAAAAAAGTGCACTGAATTTATTAGACAAAC<br>TTACGAATGCTTAACTTCTTTACACAGCATAGGTGAAAATCATATTTGGGCTATTGTATACTATGAACAATTTGTAAATGTCTTAATT<br>TGATGTAAATAACTCTGAAACAAGAGAAAAGGTTTTTAACTTAGAGTAGCCCTAAAATATGGATGTGCTTATATAATCGCTTAGTTTT<br>GGAACTGTATCTGAGTAACAGAGGACAGCTGTTTTTTAACCCTCTTCTGCAAGTTTGTTGACCTACATGGGCTAATATGGATACTA<br>AAAATACTACATTGATCTAAGAAGAAACTAGCCTTGTGGAGTATATAGATGCTTTTCATTATACACACAAAAATCCCTGAGGGACAT<br>TTTGAGGCATGAATATAAAACATTTTTATTTCAGTAACTTTTCCCCCTGTGTAAGTTACTATGGTTTGTGGTACAACTTCATTCTATA<br>GAATATTAAGTGGAAGTGGGTGAATTCTACTTTTTATGTTGGAGTGGACCAATGTCTATCAAGAGTGACAAATAAAGTTAATGATGA<br>TTCCAAATTTGTGTCATTGCAGTACTAAATCTATTTTATATTGTAAAAACCTAACAAAACAGGCTTTTCTTTCATCAGGATTGGCTGT<br>TTTGTTGTACAGATAATTTCATGGTGTTTCTTTTACACTTGTTTATTCTCGCTGCCTCCTGAGATTTAGTACTAATGTAAGGAAGGCA<br>TTTTAATTACCATTCTTGTTACTGAATTTATATAGGATATAT
MUT-ATP6AP2	ATGTTACCTGTGCCAGAATTAGAAAAGGGGGTTGGAAATTGGCTGTTTTGTTAAAATATATCTTTTAGTGTGCTTTAAAGTAGATAG<br>TATACTTTACATTTATAAAAAAAAATCAAATTTTGTTCTTTATTTTGTGTGTGCCTGTGATGTTTTTCTAGAGTGAATTATAGTATTGA<br>CGTGAATCCCACTGTGGTATAGATTCCATAATATGCTTGAATATTATGATATAGCCATTTAATAACATTGATTTCATTCTGTTTAATG<br>AATTTGGAAATATCGTGACTAAGAAATGTAAAACATTTAGAATAGCTCGTGTTATGGAAAAAAGTCGTGACTATTTATTAGACAAAC<br>TTACGAATGCTTAACTTCTTTACACAGCATAGGTGAAAATCATATTTGGGCTATTGTATACTATGAACAATTTGTAAATGTCTTAATT

TGATGTAAATAACTCTGAAACAAGAGAAAAGGTTTTTAACTTAGAGTAGCCCTAAAATATGGATGTGCTTATATAATCGCTTAGTTTT
GGAACTGTATCTGAGTAACAGAGGACAGCTGTTTTTTAACCCTCTTCTGCAAGTTTGTTGACCTACATGGGCTAATATGGATACTA
AAAATACTACATTGATCTAAGAAGAAACTAGCCTTGTGGAGTATATAGATGCTTTTCATTATACACACAAAAATCCCTGAGGGACAT
TTTGAGGCATGAATATAAAACATTTTTATTTCAGTAACTTTTCCCCCTGTGTAAGTTACTATGGTTTGTGGTACAACTTCATTCTATA
GAATATTAAGTGGAAGTGGGTGAATTCTACTTTTTATGTTGGAGTGGACCAATGTCTATCAAGAGTGACAAATAAAGTTAATGATGA
TTCCAAATTTGTGTCATTGCAGTACTAAATCTATTTTATATTGTAAAAACCTAACAAAACAGGCTTTTCTTTCATCAGGATTGGCTGT
TTTGTTGTACAGATAATTTCATGGTGTTTCTTTTACACTTGTTTATTCTCGCTGCCTCCTGAGATTTAGTACTAATGTAAGGAAGGCA
TTTTAATTACCATTCTTGTTACTGAATTTATATAGGATATAT
